# Glutamic acid reshapes the plant microbiota to protect plants against pathogens

**DOI:** 10.1186/s40168-021-01186-8

**Published:** 2021-12-20

**Authors:** Da-Ran Kim, Chang-Wook Jeon, Gyeongjun Cho, Linda S. Thomashow, David M. Weller, Man-Jeong Paik, Yong Bok Lee, Youn-Sig Kwak

**Affiliations:** 1 RILS, Gyeongsang National University, Jinju, 52828 Republic of Korea; 2 Division of Applied Life Science (BK 21 plus) and IALS, Gyeongsang National University, Jinju, 52828 Republic of Korea; 3 US Department of Agriculture, Agricultural Research Service, Wheat Health, Genetics and Quality Research Unit, Pullman, WA 99164-6430 USA; 4 College of Pharmacy, Sunchon National University, Suncheon, 65980 Republic of Korea; 5 Department of Plant Medicine, Gyeongsang National University, Jinju, 52828 Republic of Korea

**Keywords:** Microbiome engineering, Glutamic acid, *Streptomyces*, Phytobiome

## Abstract

**Background:**

Plants in nature interact with other species, among which are mutualistic microorganisms that affect plant health. The co-existence of microbial symbionts with the host contributes to host fitness in a natural context. In turn, the composition of the plant microbiota responds to the environment and the state of the host, raising the possibility that it can be engineered to benefit the plant. However, technology for engineering the structure of the plant microbiome is not yet available.

**Results:**

The loss of diversity and reduction in population density of *Streptomyces globisporus* SP6C4, a core microbe, was observed coincident with the aging of strawberry plants. Here, we show that glutamic acid reshapes the plant microbial community and enriches populations of *Streptomyces*, a functional core microbe in the strawberry anthosphere. Similarly, in the tomato rhizosphere, treatment with glutamic acid increased the population sizes of *Streptomyces* as well as those of Bacillaceae and Burkholderiaceae. At the same time, diseases caused by species of *Botrytis* and *Fusarium* were significantly reduced in both habitats. We suggest that glutamic acid directly modulates the composition of the microbiome community.

**Conclusions:**

Much is known about the structure of plant-associated microbial communities, but less is understood about how the community composition and complexity are controlled. Our results demonstrate that the intrinsic level of glutamic acid in planta is associated with the composition of the microbiota, which can be modulated by an external supply of a biostimulant.

**Video Abstract**

**Supplementary Information:**

The online version contains supplementary material available at 10.1186/s40168-021-01186-8.

## Introduction

The plant microbiome includes associated microorganisms residing above and below ground, and inside or outside of plant tissues [1]. Plant microbiomes play a critical role in plant development and health [2–4], and it is reasonable that maintenance of a healthy microbiome would promote growth and crop yield in agricultural systems [5, 6]. This ecological and functional integration of the plant and its microbiome are encompassed within the holobiont, the assemblage of the host, and the other species living in or around it, which together form a discrete ecological unit [7, 8]. Moreover, complex microbial populations reside in association with all plant tissues, implying that the initial phases of colonization, as well as subsequent microbe-microbe interactions, selectively influence the structure of the microbiome [9–12] and that the core microbial community has a vital role in the overall microbiome stability and the fitness of the host [13, 14].

Throughout plant development, the structure of the associated microbial community has a significant impact on plant health, especially in disease prevention by beneficial microbes such as *Bacillus*, *Pseudomonas*, and *Streptomyces* [10, 15, 16]. A biostimulator can be defined as a substance or microorganism applied to a host with the effect of enhancing micro-nutrient uptake or abiotic stress tolerance to improve crop quality [17]. Kauffman et al. defined the first plant biostimulators as “materials, other than fertilizers, that promote plant growth when applied in low quantities” [18]. The term biostimulator is frequently used in the scientific literature, and collectively refers to substances with biological functions [19, 20]. Biostimulators are known to affect both plants and microbes [21–23] and, like prebiotics, can influence the host indirectly through their effects on the associated microbiome [24].

Trends in plant microbiome studies have approached microbiome engineering with the goal of improving plant health and productivity [25] via either a top-down or a bottom-up approach [14]. The top-down approach refers to manipulation of environmental and physicochemical conditions to select the desired biological process [26, 27]. This top-down design involves relatively macro-scale processes resulting in microbiome engineering. Conversely, bottom-up approaches link molecular and biochemical characteristics and relatively micro-scale processes with precise mechanisms in the interaction [27]. Consequently, these applied to microbiome-associated phenotypes [27]. In humans or animals, they can be facilitated by prebiotics, a terminology devised in 1995 by Gibson and Roberfroid [28]. Such prebiotics may selectively influence the gut microbiome [29–32] or move to other organs through the blood, directly influencing animal health [33]. In plants, a similar role is played by biostimulators [20], defined as substances applied to plants with the aim to enhance nutrition efficiency, abiotic stress tolerance, and/or crop quality traits, regardless of nutrient content [34, 35]. In recent years, the terminology has been extended in scope from screening substances to understanding their mode-of-action [19, 36]. Thus, humic acid, fulvic acid, and seaweed extract were identified as biostimulators that enhanced tolerance against abiotic stress, promoted plant growth, and improved soil quality [34, 36]. Such biostimulators may contain plant hormone-like compounds or activate hormone activities as their mode-of-action but so far, the mechanisms underpinning biostimulator function are poorly understood [36].

We previously reported *Streptomyces globisporus* biocontrol strains S4-7, SP6C4, and SF7B6 associated with strawberry rhizosphere, pollen, and flowers, respectively, and recovered strain TFH56, with antifungal activity and the same genome structure, from tomato pollen [16, 37–39]. We also observed the collapse of the strawberry anthosphere microbial community structure coincident with the aging of plants [16]. In particular, the loss of diversity and reduction in population density of *Streptomyces globisporus* SP6C4, a core microbe, was negatively correlated with the onset of two major anthosphere diseases, gray mold (*Botrytis cinerea*) which causes brown spots on flower petals, and blossom blight (*Cladosporioides* sp.), which appears as fuzzy gray mycelium on flower pistils and stamens. We then hypothesized that a specific plant metabolite could be amended to rebuild the microbial community structure to maintain the health of the plant. Here, we propose that glutamic acid configures the microbial community and modulates the abundance of *S*. *globisporus* SP6C4.

## Results

### Microbiome collapse and disease

**Fig. 1:**
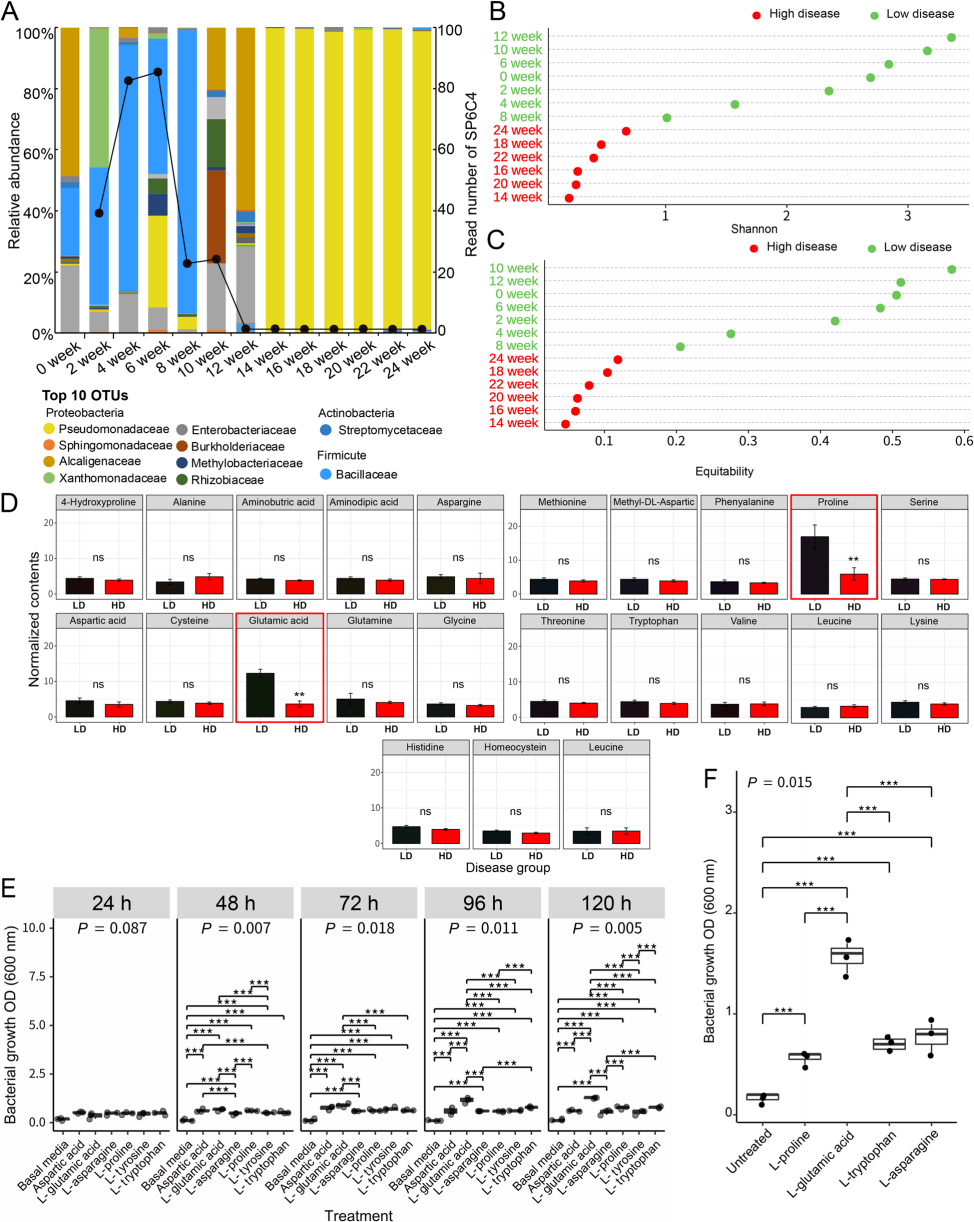
Amino acid content of strawberry flower petal tissues. Microbial community collapse in the strawberry anthosphere (Kim et al. 2019). **A** Microbiome diversity of strawberry flowers (*n* = 9, 13 independent experiments). Strawberry (cv. Maehyang) flowers were collected from week 0 (Nov. 2013) to week 24 (Apr. 2014). Top 10 phyla at the family level based on the Silva database (http://www.arb-silva.de/) and black line presented the SP6C4 OTUs number by 98% similarity. **B**, **C** ASVs alpha diversity over time according to Shannon’s diversity and equitability indices relative to gray mold disease incidence. **D** Content of 23 amino acids in strawberry flower petals (*n* = 3, 8 independent experiments). Normalized concentrations of amino acids were compared by independent *t* test (*P* value < 0.05). Black and red bars indicate periods of low and high disease incidence, respectively. **E** Growth of *S*. *globisporu*s SP6C4 in basal medium supplemented with amino acids. A bacterial suspension (100 μl, OD_600nm_ 0.02) was inoculated into basal medium supplemented with amino acids (2%) in a 96-well plate and incubated on an orbital shaker at 150 rpm and 28 °C for 120 h (*n* = 3, 3 independent experiments). **F** Bacterial growth with 2% amino acid amendments. Box-and-whisker plot presenting boundaries of the rectangles indicate the 25th and 75th percent and the horizontal bars indicated the median. A Kruskal-Wallis rank-sum test and stars indicate Tukey’s HSD test, statistically significant differences among treatments (*P < 0.05, **P < 0.01, ***P < 0.001)

The microbial community of strawberry flowers shifts throughout the growing season from one of high diversity (weeks 0–12) to one of low diversity (weeks 14–24), a pattern coincident with the loss of *S*. *globisporus* SP6C4, which we consider to be a core member of the flower microbial community [16]. Here, we recalculated strawberry flower microbial population data to identify the top 10 phyla and the diversity of the microbial community throughout the growing season (Fig. 1A) of strawberry plants grown in a commercial climate-controlled greenhouse (Table S1). We found that populations of the core microbe *S*. *globisporus* SP6C4 decreased at week 8 and could no longer be detected in flower samples after week 14, at which time the community had collapsed (Fig. 1A). In contrast, the incidence of gray mold disease increased from weeks 14–24, coincident with the onset of disease in plants exposed to the pathogen (Fig. 1B, C). These patterns indicated that the microbial community structure changed with the age of the plant or stage of blossoming, and that the collapse of the community included loss of core microbiota.

### Effect of plant exudates on the core microbe

Petal and ovary exudate samples were analyzed for amino acids, organic acids and soluble sugars (Fig. 1D and Fig. S1). Amino acids in the petals did not differ significantly throughout periods of low and high disease incidence except for glutamic acid and proline, the content of which decreased significantly during the period of microbial community collapse and high gray mold disease incidence. In contrast, the content of the 23 amino acids in the flower ovary did not vary regardless of disease incidence (Fig. S1A). Similarly, the content of soluble sugars and organic acids in petals and ovaries remained consistent between periods of low and high disease (Fig. S1B, C, D). These findings suggest that glutamic acid and proline have a key role in maintaining the density of the core bacterium SP6C4 in the anthosphere. Biolog plates PM1 (Phenotype microarray) and PMB3 were used to identify carbon (PM1) and nitrogen (PMB3) sources influencing the core strain. *S*. *globisporus* SP6C4 grew equally well on the carbon substrates (Fig. S2A and Table S2), but growth on nitrogen substrates increased markedly on l-tyrosine, l-proline, l-aspartic acid, v-cysteine, agmatine, and l-glutamic acid (OD_590_ ≥ 0.4) (Fig. S2B and Table S3). However, only l-glutamic acid influenced the growth of *S*. *globisporus* SP6C4 in both the amino acid analysis of flowers and the Biolog plates. Bacterial growth was further evaluated with the four amino acids l-glutamic acid, l-proline, aspartic acid, and l-tyrosine, with basal medium and l-asparagine as negative controls and l-tryptophan as a positive control. SP6C4 did not grow on unamended basal medium, but growth (OD_600_) for 120 h was 0.6 for l-asparagine, 0.7 for aspartic acid, 0.8 for l-proline, 0.8 for l-tryptophan, and 1.3 for l-glutamic acid (Fig. 1E). As additional assessment, growth was evaluated in basal medium supplemented with each amino acid at 0.02% (1.3 μM), 0.2% (13 μM), and 2% (130 μM) (Fig. 1F and Fig. S2C, D). Regardless of concentration, l-glutamic acid had the greatest effect on the growth of SP6C4.

### Glutamic acid modulates the strawberry anthosphere microbiota

We next evaluated whether l-glutamic acid can modulate the structure of the strawberry anthosphere microbial community. Each of three treatments (untreated control, l-glutamic acid, or l-asparagine at a final concentration of 2%, pH 6.5) was sprayed for 1 min per plot at 2-week intervals during January and February, 2018. Sequencing analyses performed on an Illumina MiSeq platform yielded a total of 3,307,450 reads (Table S4). The Silva database (http://www.arb-silva.de/; Silva_SSU_r132) [40, 41] was used to identify the community members associated with the shift in overall community structure in response to glutamic acid. A divisive amplicon denoising algorithm2 (DADA2) predicted a total of 162 amplicon sequencing variants (ASVs), with Enterobacteriaceae dominant from weeks 2–8 in the untreated control (93.1, 91.9, 94.3, and 74.9%), in the l-asparagine treatment (95.9, 80.6, 55.9, and 94.3%), and from weeks 2–4 in the l-glutamic acid treatment (99.8 and 73.5%). Pseudomonadaceae were the second most abundant ASV, followed by Moraxellaceae (Fig. S3A, C). Enrichment of Streptomycetaceae occurred only in the l-glutamic acid treatment during weeks 6 and 8, accounting for 99.9 and 99.9% of the community. These results are indicated by a change in the color of the heatmap from purple to yellow (Fig. S3B). Compared to the relative abundance of ASVs calculated as log2 ratios in Metacoder, Streptomycetaceae had a log2 ratio value of 3 only upon treatment with l-glutamic acid (Fig. S4).

**Fig. 2:**
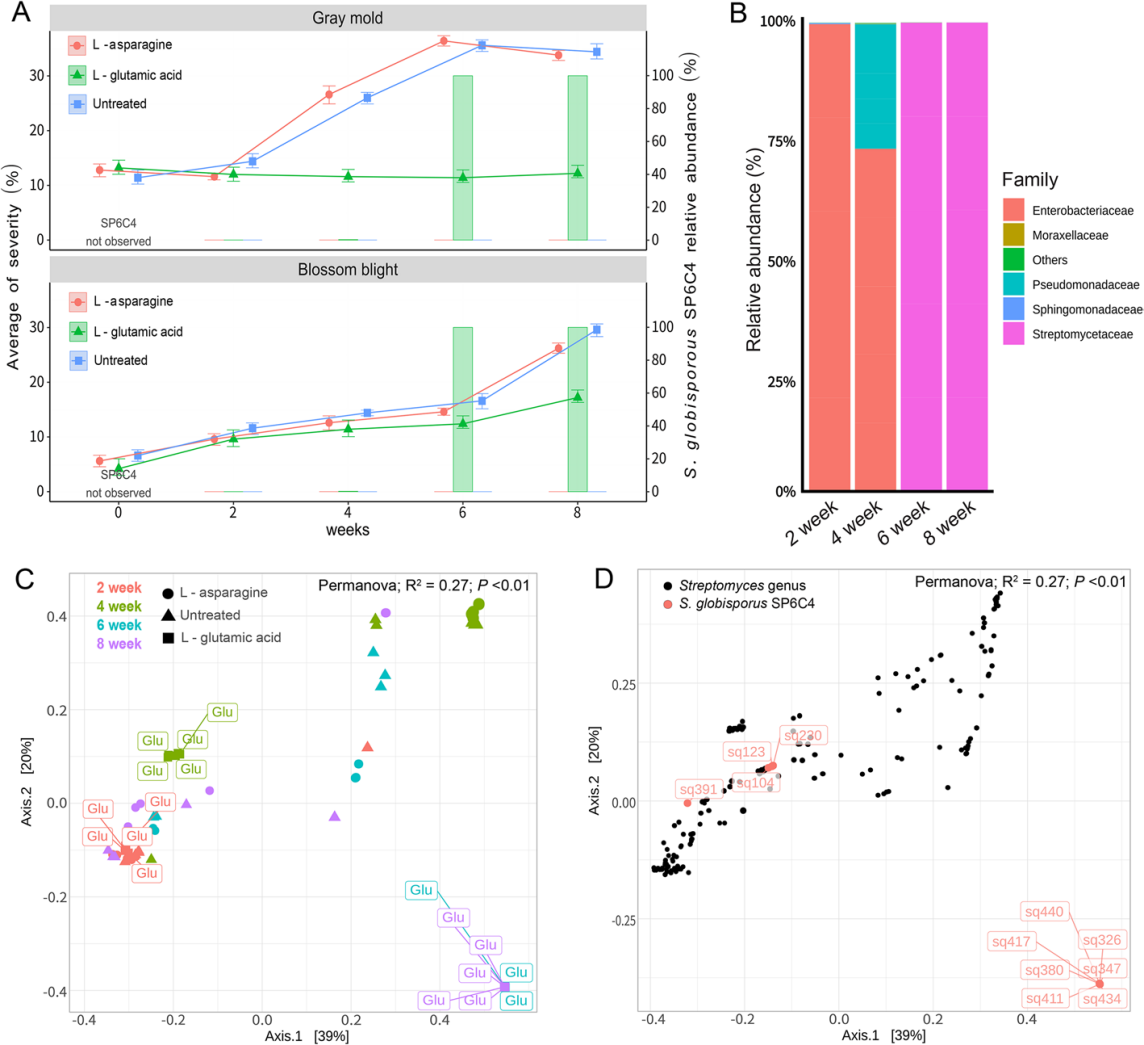
Microbial diversity in the strawberry flower is shifted by different amino acid treatments. Samples were collected from December 2017 to February 2018 and amino acids were sprayed from Jan, 2018 to Feb, 2018 (weeks 4, 6, and 8). Each treatment included five plots of 100 plants (*n* = 5, 12 independent experiments). **A** Strawberry gray mold disease and blossom blight disease severity % with *Streptomyces globisporus* SP6C4 relative abundance, which OTUs was similarity over than 98% (*n* = 100, each replication was independent between treatment). **B** Sequencing of the microbes associated with strawberry flowers with 2% l-glutamic acid treated (*n* = 5, 12 independent experiments). Taxonomic assignment was conducted at the family level in Silva database (http://www.arb-silva.de/) with a similarity cutoff of 97% confidence. **C**, **D** PCoA plots of beta diversity (Bray-Curtis distance); each sample was vectorized to spatial position and dark circles in **e** covered OTUs overlapping those of *Streptomyces globisporus* SP6C4 (identities > 98%)

The microbial communities of l-glutamic acid-treated flower samples (weeks 6 and 8) were also clearly distinguished. The plot with *Streptomyces* ASVs showed that the population of the core microbe represented more than 90% of the microbial community in l-glutamic acid-treated samples on weeks 6 and 8 (Fig. 2B). Also, principal coordinate analysis (PCoA) data clearly distinguished groups, one that consisted of the untreated control and the l-asparagine treated samples (weeks 2 and 4) and the other, comprised of l-glutamic acid-treated flowers (weeks 6 and 8) (Fig. 2C, D). PCoA analysis presented 11 ASVs with sequence identity to *S*. *globisporus* SP6C4 of greater than 98% (Fig. 2C, D).

The population of SP6C4 was determined by qPCR (quantitative polymerase chain reaction) with primers for the SP6C4-specific *lanM* lantipeptide biosynthesis gene [39]. On flowers sprayed with l-glutamic acid, the density of SP6C4 was greater than 10^5^ copy/g of flower, but fewer than 10^4^ copy/g of flower were detected at 8 weeks on the untreated control and on flowers sprayed with l-asparagine (Fig. S5A). These results were transformed to a bubble plot that verified that l-glutamic acid increased the population size of strain SP6C4 on strawberry flowers and was correlated with the low disease incidence but high *lanM* copy number from week 4 to week 8 compared to the untreated control and the l-asparagine treatment (Fig. S5B).

### Tomato rhizosphere microbiota composition was shifted by glutamic acid

**Fig. 3:**
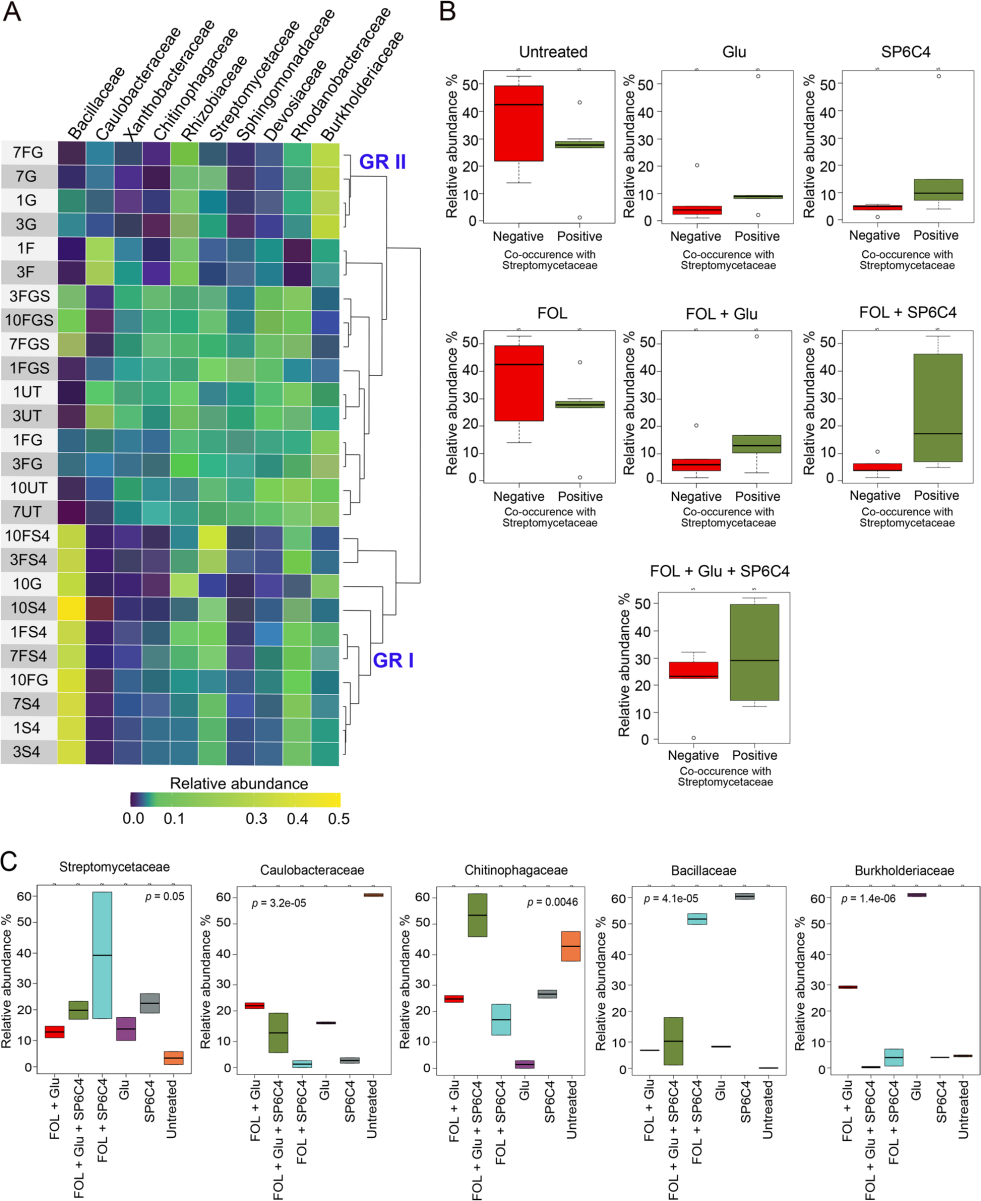
ASVs abundance at the family level of two different co-related groups relative to the *lanM* (grisin) copy number. Tomato plants were cultured in a plant growth chamber for 10 weeks (16 h: Light, 25 °C, 8 h: Dark, 22 °C). l-glutamic acid 50 ml (5 μg/ml) was drenched at 1 and 2 weeks and 30 ml of *S*. *globisporus* SP6C4 (10^7^ cfu/ml) in 0.1% methylcellulose was added at the base of the plant at 4 weeks. At 5 weeks, conidia of *F*. *oxyporum* f. sp. *lycopersici* (FOL, 10^5^ cfu/ml) were inoculated. Rhizosphere samples were collected at 1, 3, 7, and 10 weeks and each treatment had 5 plants (*n* = 5, 26 independent experiments). **A** Distribution heatmap of microbial abundance ordered by hierarchical clustering with 16S rRNA. Heatmap color (purple to yellow) corresponds to ASVs abundance from low to high. The tree on the right was created by the Minkowski distance method. **B** Negative group: Caulobacteraceae, Chitinophagaceae, Positive group: Bacillaceae, Burkholderiaceae. Boxes present average relative abundance with standard error of ASVs in each of six treatments at 3 week (Independent sample *t* test: untreated, *P* = 0.16; glutamic acid, *P* = 0.38; SP6C4, *P* = 0.14; FOL, *P* = 0.17, FOL + Glu, *P* = 0.29; FOL + SP6C4, *P* = 0.12; FOL + Glu + SP6C4, *P* = 0.35). **C** Variation in abundance of the major ASVs relative to the core microbe and wilt disease sensitivity after treatment with L–glutamic acid (5 μg/ml) and *Fusarium oxysporum* f. sp. *lycospersici* (conidia 10^5^ cfu/ml). The relative abundance at 1 and 3 weeks before disease inoculation (*n* = 2, 6 independent replications) and numeric values are visualized with NOI-seq package (version 3.10). Box-and-whisker plot presenting boundaries of the rectangles indicate the 25^th^ and 75^th^ percent and the horizontal bars indicated the median

The anthosphere of strawberry has only a few species in the microbial community. Therefore, to investigate changes over time in the microbial community structure of rhizosphere, tomato samples were collected 1, 3, 7, and 10 weeks after treatment except for the *Fusarium oxysporum* f. sp. *lycopersici* (FOL) only treated samples, none of which survived for 10 weeks. Total sequencing read numbers were 5,863,545 (Table S5) and the number of ASVs counted was 3247. All sequences were compared with taxa in the Silva database (Silvar_SSU_r138) at the ASV level, and the top 10 families present in greatest relative abundance were visualized (Fig. S6A). Bacillaceae, Burkholderiaceae, Streptomycetaceae, Rhodanobacteraceae, Rhizobiaceae, Devosiaceae, Caulobacteraceae, Sphingomonadaceae, Xanthobacteraceae, and Chitinophagaceae each represented more than 5% of the community. PCoA results indicated that over time, the rhizosphere community of glutamic acid-treated tomato became similar to the SP6C4-treated tomato rhizosphere community. In contrast, the untreated control tomato rhizosphere microbiota was clearly distinguished from both the SP6C4 and the glutamic acid treated tomato microbial population (Fig. S6B). Heatmap analysis revealed changes in the rhizosphere microbial community structure in relation to treatments, which were divided into three distinguishable clusters (Fig. 3A). The first cluster was enriched in Bacillaceae by treatment with strain SP6C4 or SP6C4+FOL (group I; GR I); treatment with l-glutamic acid alone led to enrichment of Burkholderiaceae (group II; GR II); and the third cluster included non-enriched members of the community in response to treatments (Fig. 3A). Co-related patterns within the tomato rhizosphere microbiome among the treatments that survived FOL treatment were analyzed by using Spearman’s algorithm (R version 3.4.4) to create a rank co-related pattern (Spearman’s ρ > 0.8) that represented either positive or negative relationships to the core microbe. Based on the Spearman’s co-related results, we analyzed the abundance of SP6C4 in both the positive and the negative ASV clusters. Streptomycetaceae, Burkholderiaceae, and Bacillaceae were identified as positively related taxa that were co-increased in density by SP6C4 or l-glutamic acid treatment. In the negatively related clusters, Caulobacteraceae, Chitinophagaceae, Devosiaceae, Rhizobiaceae, and Xanthobacteraceae were identified as important taxa. At week 3, before inoculation with FOL, communities of the untreated control and those after treatment with FOL had more negative clusters than positive ones but the finding was not significant (Fig. 3B). These results indicated a limitation to microbial composition analysis with only two clusters. Therefore, we also analyzed the relative abundance (RA) of the positively related taxa in both the positive (Bacillaceae and Burkholderiaceae) and negative (Caulobacteraceae, and Chitinophagaceae) clusters. Before treatment with FOL, Streptomycetaceae were present at 40% RA in the FOL + SP6C4 treatment. The RA of Bacillaceae was 60% in the SP6C4-treated rhizosphere and that of Burkholderiaceae was the greatest (60%) in the rhizosphere of l-glutamic acid-treated tomatoes. Interestingly, in the untreated control plants and the negative clusters, the most abundant microbes were Caulobacteraceae (60%) and Chitinophagaceae (45%) (Fig. 3C). Taken together, the microbial community structure in the tomato rhizosphere was affected by introduction of strain SP6C4 or l-glutamic acid. The introduction of the core microbe, SP6C4, enriched Bacillaceae, and drenching with l-glutamic acid increased the density of Burkholderiaceae in the rhizosphere. The findings indicated that SP6C4 and l-glutamic acid have different modulating effects on the rhizosphere microbiome community.

### Effect of glutamic acid on strawberry flower disease suppression

**Fig. 4:**
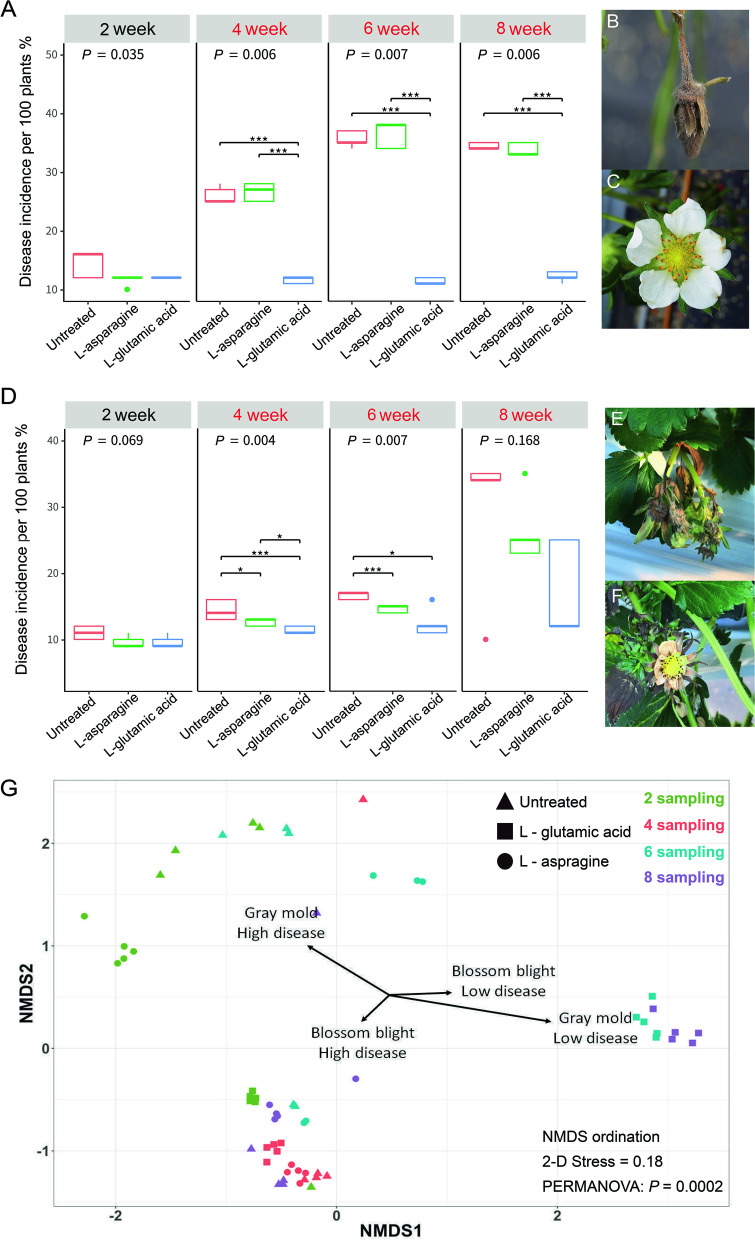
Changes in microbial community structure coincident with amino acid treatment and correlation with disease occurrence. Plants in the greenhouse (8 m wide × 82.5 m length; 660 m^2^) were arranged in 7 longitudinal rows, 3 of which were untreated; 2% l-asparagine and 2% l-glutamic acid were applied by sprayer (HP-2010, Korea, 1.5 L discharge capacity/min) at intervals of 2 weeks. Mean disease incidence of 5 independent box plots; Box-and-whisker plot presenting boundaries of the rectangles indicate the 25^th^ and 75^th^ percent and the horizontal bars indicated the median and calculated by Tukey’s HSD (*P < 0.05, **P < 0.01, ***P < 0.001, ****P < 0.0001. Red letters in the legend indicate when amino acids were sprayed. **A** Gray mold disease incidence, **B** strawberry flower infected by *Botrytis cinerea*, with brown spots on petals and fruits covered by conidia, **C** uninfected healthy flower. **D** Blossom blight disease incidence. **E**, **F** Strawberry flower and fruit infected by *Cladosporium cladosporioides* with dark leaf and calyx*.* **G** Ordination plot of NMDS analysis based on microbial diversity and relative abundance of ASVs (*n* = 5, 12 independent experiments). Four vectors correspond to disease incidence variables (for each block, *n* = 100 plants, 5 blocks represent independent experiments). For gray mold, low disease was < 15% and high disease incidence was 16 to 30%. Low incidence of blossom blight was < 20% and high incidence was 21 to 30%

We assessed the effect of l-glutamic acid on the occurrence of gray mold, blossom blight, and the density of strain SP6C4 in strawberry flowers. Disease incidence (DI) was evaluated at 2-week intervals. At the same time, l-glutamic acid and l-asparagine were sprayed three times, at 2-week intervals, from week 4 to week 8 in a strawberry greenhouse (Fig. S7). Gray mold DI from week 0 to week 4 remained relatively low (10–16%) regardless of treatment. At 6 weeks, the untreated control presented a DI of 16.6%; the DI in the l-asparagine-treated plot was 16%, and the DI in the plot treated with l-glutamic acid was significantly lower, at 12.4%. At week 8, the DI in the untreated control increased to 34% but that in the plot sprayed with l-glutamic acid was maintained below 17% (Fig. 4A, B, C). DI values for blossom blight presented even greater differences among the treatments. At 8 weeks, 35% of flowers in the untreated control and 36% of those treated with l-asparagine developed disease symptoms, whereas fewer than 11% showed symptoms of blossom blight in the l-glutamic acid-treated plants (Fig. 4D, E, F).

The core microbe density and the disease severities (gray mold and blossom blight disease in strawberry) showed different patterns after l-glutamic acid treatment (Fig. 2A). Strawberry anthosphere microbial community structure also was influenced by gray mold and blossom blight (Fig. 4G). The effect of the amino acids on the structure of the microbial community and suppression of plant diseases was analyzed with non-metric multidimensional scaling (NMDS, Bray-Curtis distance method), to visualize the enrichment of the core microbe during disease incidence. The dispersion in NMDS indicated that the composition of the microbial community was co-related with patterns of disease occurrence, and in particular, that disease occurrence was suppressed by treatment with l-glutamic acid (Fig. 4G).

**Fig. 5:**
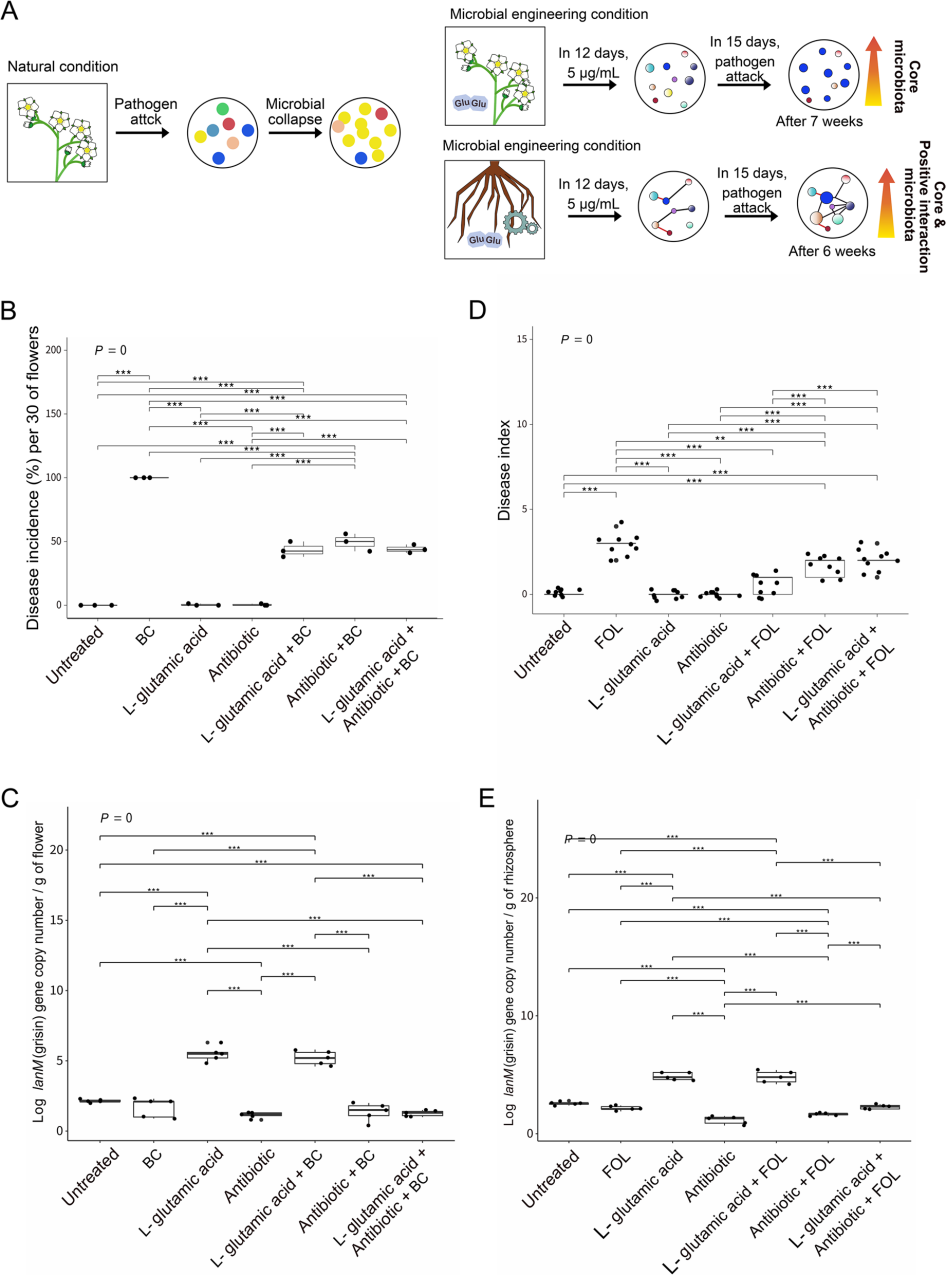
Microbial engineering with l-glutamic acid (5 μg/ml) for control of plant disease. Strawberry plants were chilled at – 2 °C for 1 month and transplanted into plastic pots (10 cm diam.) with autoclaved soil. **A** Microbial engineering experiment design in the strawberry anthosphere and tomato rhizosphere. At strawberry flowering (12 days), l-glutamic acid (5 μg/ml) and antibiotics (erythromycin and clindamycin, 10 μg/ml each) were applied. Three days later, conidia of the gray mold pathogen *B*. *cinerea* (BC, 10^5^ cfu/ml) were sprayed onto the flower surface. **B** Infected strawberry flowers were counted to determine disease incidence (*n* = 30). **C** Copy number of the SP6C4-specific *lanM* gene (grisin) on strawberry flowers was determined by qPCR (*n* = 5, replication). Tomato grown on autoclaved soil with 0.1% Hoagland’s solution (*n* = 9 independent replicates). After 12 days, plants were drenched with 50 ml of l-glutamic acid (5 μg/ml). Six hours later, antibiotics (erythromycin and clindamycin, 10 μg/ml each) were added to the soil. At 15 days, plants were inoculated with 10 ml of FOL conidia (10^5^ cfu/ml). **D** Disease index was scored at 6 levels: 0, no symptoms; 1, slight yellowing of the lower leaves; 2, moderate yellowing of the entire plant; 3, wilted plant; 4, plants severely stunted or browning; 5, plants dead. **e** *lanM* gene (grisin) specific copy number in tomato rhizosphere by qPCR (*n* = 5, replication). Untreated control received 50 ml of 0.1% Hoagland’s solution treated flowers (sprayed) with l-glutamic acid (5 μg/ml) and antibiotics (erythromycin and clindamycin: each at 10 μg/ml). After 3 days, a stock of *B*. *cinerea* conidia (10^5^ cfu/ml) was sprayed on the flower surface. Box-and-whisker plot with boundaries of the rectangles indicating the 25^th^ and 75^th^ percentages and the horizontal bars indicating the median. Strawberry gray mold disease severity and tomato Fusarium wilt disease indices were compared by a Kruskal-Wallis rank-sum test and calculated by Tukey’s HSD (*P < 0.05, **P < 0.01, ***P < 0.001, ****P < 0.0001)

Given the dependence of the core biocontrol strain SP6C4 on glutamic acid for growth, we next tested the effect of the amino acid on disease suppression in strawberry. We established 7 treatments including an untreated control, *Botrytis cinerea* (BC) only, l-glutamic acid, antibiotics, l-glutamic acid with antibiotics, antibiotics with BC, and l-glutamic acid, antibiotics, and BC. The pathogen only treatment showed 100% disease incidence, but infection was less than 50% in the treatment with l-glutamic acid and BC (Fig. 5B). The population size of SP6C4 was increased significantly in treatments of strawberry with l-glutamic acid only or l-glutamic acid with BC (10^5^ *lanM* gene copies per g of flower) (Fig. 5C).

### Effect of glutamic acid on tomato root disease suppression

To extend our results to a more complex rhizosphere system, we evaluated the effect of glutamic acid on *Fusarium oxysporum* f. sp. *lycopersici* (FOL), a soilborne pathogen that causes Fusarium wilt disease of tomato. We established seven experimental treatments: untreated control, *S*. *globisporus* SP6C4, l-glutamic acid, FOL, SP6C4 + FOL, l-glutamic acid + FOL, and SP6C4 + l-glutamic acid + FOL (Fig. S8A). Disease severity was evaluated 7 weeks later on a scale of 0–5. Plants treated with FOL alone presented severe disease symptoms at 5 days and all of them had died by 15 days, but the plants from the other treatments had disease indices of less than 2 even at 15 days (Fig. S8B). Quantitative polymerase chain reaction (qPCR) of the SP6C4 *lanM* gene grisin in the tomato rhizosphere revealed that plants treated with the bacterial strain or even l-glutamic acid alone had more than 10^6^ *lanM* gene copies per 100 ng of soil DNA regardless of the presence of the pathogen. However, the untreated control and FOL only-treated plants had fewer than 10^2^ *lanM* gene copies, the background level (Fig. S8C). Taken together, these results showed that strain SP6C4 or l-glutamic acid alone suppressed Fusarium wilt disease in tomato and enhanced the population density of microbes carrying the *lanM* gene in the rhizosphere. Additionally, at 15 days, both shoot length and shoot weight were significantly reduced by treatment with FOL, but the damage was lessened by treatment with strain SP6C4 or l-glutamic acid (Fig. S8D, E). When the enrichment of endogenous *Streptomyces* by glutamic acid was eliminated by treatment with antibiotics (erythromycin and clindamycin, 10 μg/ml each), which is active specifically against the bacteria, tomato was no longer protected against the FOL pathogen (Fig. 5D).

The population size of SP6C4 in the rhizosphere of tomato plants treated with l-glutamic acid only or l-glutamic acid + FOL, as measured by qPCR with the *lanM* grisin gene, was 10^5^ gene copies/g of rhizosphere soil. However, the untreated control, FOL, antibiotics, antibiotics + FOL, or l-glutamic acid + antibiotics + FOL-treated plants showed significantly lower density of the core microbe, with only 10^3^ *lanM* gene copies/g of rhizosphere soil (Fig. 5E). These results indicated that the SP6C4 population in the endogenous microbiota was enriched by exogenous l-glutamic acid. Collectively, we interpret these results to indicate that glutamic acid increased the density of *Streptomyces*, the functional core microbe, which suppressed the fungal pathogen.

## Discussion

The microbiome extends the genetic and physiological capacity of the host, contributing to its growth and well-being by providing ecological services and protection from biotic and abiotic stresses. Much as the animal gut microbiome can be influenced by probiotics, diet [32, 42, 43], or prebiotics [44–46] with the potential to engineer its composition or activity, so also is the structure of plant microbiome community responsive to the types and amounts of metabolites present in plant exudates secreted into the rhizosphere [47, 48]. The plant developmental stage and genotype influence the microbiome community structure as well as the root architecture and chemistry, which have a significant impact on microbiome composition [49]. The quality and quantity of root exudate directly impact rhizosphere microbiome assembly.

A calcium-dependent antibiotic such as a lipopeptide of *Streptomyces coelicolor* is regulated by tryptophan, and the production of other secondary metabolites such as rapamycin and pristinamycin also are controlled in *Streptomyces* spp. by the quality and quantity of the nitrogen sources [23, 50]. In addition, exogenous glutamate improved streptolydigin production of *Streptomyces lydicus* [51]. The glutamate uptake system is encoded by GluR-GluK, a two-component system that plays roles in physiological metabolism of *Streptomyces* spp. [52]. However, the influence of these compounds is poorly understood in relation to the molecular mechanisms of the plant microbial community.

We have shown here that glutamic acid, either secreted by the plant or added exogenously, functions as a prebiotic and is a key tool in a bottom-up model of plant microbiome engineering [14, 53], increasing the population density of *Streptomyces* as a core member of the microbial community. In both the anthosphere of strawberry, with its very simple microbiota, and in the complex rhizosphere microbiota of tomato, glutamic acid initiated a cascade resulting in reconfiguration of the microbiota and enrichment of *Streptomyces* in the community. A similar conclusion was reached in a study with *Sphingomonas melonis*, which accumulated across generations, resulting in disease suppressive ability [54]. Of note is that as a consequence of this process in tomato, both foliar and root pathogens were controlled. On unrelated plant species, it is not uncommon for chemicals applied to plants to induce systemic resistance, but with glutamic acid the effect was not due to the induction of resistance through either the ethylene/jasmonic acid or the salicylic acid pathway. We were surprised by the extent of modification of the two microbiota by the addition of a single chemical. However, some substrates are preferentially metabolized by microbes [44] and can selectively engineer the composition or activity of entire microbial communities, influencing the health of the host [45, 55], much as when diet affects the composition of the gut microbiome [29, 32, 43, 55]. Previous research reported that the d-type amino acid has a broad effect on plants or microbiota [56]. However, in our system, we could not detect d-glutamic acid in strawberry flower exudates, and d-glutamic acid did not affect the growth of SP6C4 based on Biolog PM assays. Therefore, we focused on l-glutamic acid as a critical amino acid to enhance the core microbe. Our results indicate that glutamic acid functions directly as a link to the microbiota; it directly affected the microbiome community structure and engineered it to suppress disease incidence. Moreover, the fact that glutamic acid did not activate host plant resistance mechanisms suggests that it may provide insight into evolutionary and functional relationships between the plant and its microbiota. Glutamic acid, in particular, is metabolized by *Streptomyces* as sole source of carbon and nitrogen, favoring vigorous growth [57], which may help to explain its effect on the plant-associated communities we observed in this study. Glutamic acid (10 mg/l) applied exogenously improved antibiotic production and the gene expression of *gluABCD*, which encodes the glutamate uptake component in *Streptomyces* spp. [52]. In this report, our finding supported the idea that glutamic acid supplied to core microbiota is a nutritional factor similar to a biostimulant.

How did the relationship of the host and the microbiome evolve? Perhaps the answer can be found in the biological function of the microbiota. Plants are constantly exposed to changing environmental forces that also act to shape the microbiota, but at the same time, the microbiota community structure is flexible and capable of buffering the impact of the environment on the host. In our experimental system, the cultivation of strawberries is a difficult and tremendous time-consuming process. Therefore, most of our experiments were conducted in commercial farm greenhouses and the experiments were performed for 5 years. Under these conditions in the anthosphere, the microbial community has a simpler ASV structure compared to that of a rhizosphere microbial community, and it would be easy to change the beta-diversity of the of strawberry ASVs. Therefore, each year, the anthosphere microbial community would be changed due to the addition or subtraction of ASVs in response to environmental factors. However, the core strain (SP6C4) always responded to glutamic acid treatment [16]. The core strain (SP6C4) also is an endogenous inhabitant of natural strawberry, which harbors a *lanM* (SP6C4 specific detection maker) gene copy number of about 10^4^/g of sample as a basal level [39]. Strawberry flowers may also have a certain endogenous SP6C4 population. However, populations below (10^5^ cfu/g) are too low to enhance plant growth and protection [58, 59].

The community structure and abundance of the plant microbiota are influenced by the amount of glutamic acid available via exudates or by exogenous delivery. Given that glutamic acid is naturally present in host exudates, it would seem that plants already have the potential to engineer protective microbiota themselves. Glutamic acid is an essential and vital nutrient element for certain microbes and microbiota communities to function as biostimulators. Anthosphere microbiota responded sensitively to exogenous glutamic acid, especially the particular bacterium that became a highly dominant strain in the presence of the relatively simple anthosphere microbiota diversity. In contrast, several bacterial families in the rhizosphere were increased in abundance by the glutamic acid treatment. These findings are consistent with the fact that the rhizosphere microbial community is a more complex system and suggest that more sophisticated and in-depth network research is required. We suggest that glutamic acid acts as an important factor in the plant holobiont concept. Thus, with better understanding of the relationship between plant exudates and microbiome assembly, it may be possible to develop crops that can recruit their own microbiota to better withstand pathogen attack.

These approaches are exactly performed by many types of concepts as microbiome modulated, that is inoculated microbial has shift on distributes of plant microbiome to improved or restored [17]. While our results indicate clearly that glutamic acid is a powerful mediator of the structure of the plant-associated microbiome, much remains to be known about how it interfaces with the complex metabolic and signaling exchanges among microbes and their plant hosts. There has been considerable progress in recent years toward elucidating the structure and function of plant-associated microbial communities, but new approaches are needed to reveal how the composition of the community and its function are controlled. Based on the results of this study, we propose that glutamic acid configures the microbial community and modulates the composition of a core microbiome that benefits the plant by influencing such agronomic metrics as crop quality and yield.

## Conclusion

In this study, we show that glutamic acid functions as a direct link between the plant host and its microbiome. In strawberry, endogenous glutamic acid in the anthosphere was correlated with the presence of the core microbe *Streptomyces globisporus* SP6C4 and control of gray mold disease. Symptoms occurred when the concentration dropped below a threshold level, and application of glutamic acid restored both the population of the core microbe and disease control. Similarly, application of glutamic acid engineered the microbiome of tomato to include *S*. *globisporus* and provided control of *Fusarium* wilt on the roots. The response to treatment with glutamic acid suggests that it affected the microbiota community directly and may provide critical clues to the possibility of engineering beneficial plant microbiota.

## Materials and methods

### Strawberry sampling

Strawberry plants (*Fragariae x ananassa* cv. Meahyang) were cultivated in a high-bed greenhouse in Jinju, Republic of Korea (34° 59′ 35.2′′ N 128° 02′ 50.3′′ E). Strawberry flowers (*n* = 15–20 per sample) were selected at random for analysis of nitrogen, carbon, and organic acid concentrations at 2-week intervals from September, 2013 to January, 2014. At each sampling time, strawberry flowers were collected from among 150~180 plants in each plot. The plot has six replicates the greenhouse which were located at the edges and in the central rows.

### Chemicals and reagents for strawberry flower exudate profile analysis

In petal and ovary, samples from 2013 to 2014 were analyzed for amino acids, organic acids and soluble sugars throughout the growing season. As internal standards, 23 amino acids (AA), 17 organic acids (OA), norvaline, 3,4-dimethoxybenzoic acid, ethyl chloroformate (ECF), and methoxyamine hydrochloride were purchased from Sigma-Aldrich (St. Louis, MO, USA). *N*-Methyl-*N*-(*tert*-butyldimethylsilyl) trifluoroacetamide (MTBSTFA) was obtained from Pierce (Rockford, IL, USA). High-performance liquid chromatography (HPLC) grade toluene, diethyl ether, ethyl acetate, and dichloromethane were purchased from Kanto Chemical (Tokyo, Japan). Hydrophilic polyvinylidene difluoride (PVDF) membrane filters (Millipore Durapore®, 0.45 μm, 25 mm diameter) were purchased from Millipore Inc. (Darmstadt, Germany). All other chemicals were of analytical grade and were used as received.

### Strawberry petal and ovary samples for exudate analysis

For amino acid (AA) and organic acid (OA) analysis, 100 μg of freeze-dried petal or ovary was mixed with 10 ml distilled water, sonicated for 30 min, and filtered through a hydrophilic PVDF membrane (Millipore Durapore®, 0.45 μm, 25 mm diameter) by centrifugation at 1077*g* for 5 min. AAs and OAs in the samples were analyzed by gas chromatography-mass spectrometry (GC–MS) using an Agilent 6890 N gas chromatograph interfaced with an Agilent 5975B mass-selective detector (70 eV, electron impact mode) equipped with an Ultra-2 (5% phenyl-95% methylpolysiloxane bonded phase; 25 m × 0.20 mm i.d., 0.11 μm film thickness) cross-linked capillary column (Agilent Technologies, Palo Alto, CA, USA). The temperatures of the injector, interface, and ion source were 260, 300, and 230 °C, respectively. Helium was used as the carrier gas at a flow rate of 0.5 ml min^−1^ in the constant flow mode. Samples were loaded in the split-injection mode (10:1); the oven temperature for AA profiling was initially set at 120 °C (2 min), rose first to 240 °C at 5 °C min^−1^ then to 300 °C (3 min) at 30 °C min^−1^. The oven temperature for OA analysis was initially 100 °C (2 min), rose first to 240 °C at 5 °C min^−1^, and then to 300 °C (5 min) at 30 °C min^−1^. The mass range scanned was 50–600 u at a rate of 0.99 scans per second. In the selected ion monitoring (SIM) mode, three characteristic ions for each AA and OA were used for peak identification and quantification.

### Amino acid and organic acid profiling and pattern recognition

AA analysis was performed by using a previous method [60, 61]. Briefly, 0.5 ml aliquots from the petal or ovary were adjusted to pH ≥ 12 with 5.0 M NaOH and diluted with 0.5 ml distilled water and 0.1 μg of norvaline as internal standard. A two-phase ethoxycarbonylation (EOC) reaction was immediately conducted in the aqueous phase. The reaction mixture was then acidified (pH ≤ 2*.*0) with 10.0% sulfuric acid, saturated with sodium chloride, and subjected to extraction sequentially with diethyl ether (3.0 ml) and ethyl acetate (2.0 ml). The combined extracts were evaporated to dryness under a gentle stream of nitrogen (40 °C). The residue was reacted (60 °C, 30 min) with MTBSTFA (20 μl) and toluene (20 μl) for GC–SIM–MS (*gas chromatography–*mass spectrometry/selective ion monitoring) analysis.

For OA profiling, 0.5 ml of the petal or ovary extract was adjusted to pH ≥ 12 with 5.0 M NaOH and 0.1 μg of 3,4-dimethoxybenzoic acid was added as an internal standard. The carbonyl groups were converted to methoxime derivatives by reaction with methoxyamine hydrochloride (1.0 mg) at 60 °C for 30 min. The reaction mixture was then acidified (pH ≤ 2*.*0) with 10.0% sulfuric acid, saturated with sodium chloride, and subjected to extraction sequentially with diethyl ether (3.0 ml) and ethyl acetate (2.0 ml). After addition of trimethylamine (5 μl), the combined extracts were evaporated to dryness under a gentle stream of nitrogen at 40 °C. The residue was reacted (60 °C for 30 min) with MTBSTFA (*N-tert-*butyldimethysily-*N*-methytrifluoroacetamide, 20 μl) and toluene (10 μl) for GC–SIM–MS analysis. The concentrations of 23 AAs and 17 OAs in each petal or ovary sample were determined based on a calibration curve derived from the corresponding mean values of a control group.

### Carbon source analysis of strawberry flowers

Soluble sugars including glucose, fructose, maltose, raffinose, and sucrose were analyzed as described by Yoon et al. [62]. Flower samples (0.1 g) were homogenized in glass tubes with 6 ml of HPLC grade ethanol (80%), and incubated at 65 °C for 20 min. The supernatant fraction was collected after centrifugation at 3500 rpm for 10 min and the process was carried out three times. The pooled extracts were filtered through a 0.45 μm syringe filter and then concentrated under nitrogen. Sugar content was determined with an Agilent 1100 high-performance liquid chromatograph (HPLC) with a refractive index detector (Agilent Tech., Germany) after baseline resolution of a column (ZORBX, 4.6 × 150 mm, 5 mm particle size, Agilent Tech) at a flow rate of 1 ml/min. Samples (20 μl) were injected with 75% acetonitrile and sugar content was calculated with an internal standard.

### Carbon and nitrogen source utilization

*Streptomyces globisporus* SP6C4 was grown on MS medium (20 g mannitol, 20 g soya, 20 g agar per l) at 30 °C for 5 days. A single colony was streaked on a fresh plate and mature spores were recovered after 10 days with a sterilized cotton ball and 1 ml of ddH_2_O. After filtration, the spore concentration was adjusted to an OD_600_ nm of 2.0, mixed with 0.2% carrageenan stock solution, and incubated, 100 μl per well, in sealed plates (PM1–carbon sources and PM3B–nitrogen sources) (Biolog, Bremen, Germany) at 28 °C for 2 days. Then 10 μl of Biolog redox dye was added to each well and the intensity of color change was monitored at OD_590_ nm every 30 min for 3 h with a Synergy H1 Hybrid Multi-Mode microplate reader (BioTek, Winooski, VT, USA) [63].

### Microbial community analysis of strawberry flowers and the tomato rhizosphere

Flower samples (1 g) were transferred to fresh tubes with 30 ml of cold 1× PBS buffer (10× PBS: 8 g of NaCl, 0.2 g of KCl, 1.44 g of Na_2_HPO_4_, 0.24 g of KH_2_PO_4_ per l, pH of 7.4) and sonicated at 35 MHz for 15 s. The supernatant was filtering with 0.2 μm pore membrane syringe filter (Corning®, NY, US) and a 10 ml of syringe to detach unwanted dust and then centrifuged at 13,000 rpm for 10 min. The upper portion of the supernatant solution was gently removed by pipetting and this step was repeated twice. Finally, the supernatant was removed by centrifugation at 4000 rpm for 20 min, the pellet was suspended in 5 ml PBS and total DNA was purified from 500 μl with a Fast DNA™ Spin Kit for Soil DNA extraction (MP Biomedicals, Irvine, CA, US) according to manufacturer’s instructions, PCR reactions were conducted with 100 ng of the purified DNA and primers 27 mF (5′-gagtttgatcmtggctcag-3′) and 518 R (5′-wttaccgcggctgctgg-3′) to amplify the V1–V3 region of 16S rRNA, and a library was generated with HerculaseII Fusion DNA Polymerase and a Nextera XT Index Kit v2 (Illumina, San Diego, CA USA). Paired-end sequencing was carried out at Macrogen (Seoul, Korea) on an Illumina MiSeq platform (Illumina Inc., San Diego, CA, USA). After the primer at the 5′-end was removed and the 3′-end was trimmed, the average quality scores per sequence location was > 30, quality scores were subjected to machine learning for divisive amplicon denoising algorithm2 (DADA2) [64]. Based on the machine learning, incorrect sequences in the reads were corrected by the amplicon sequence variant (ASV) method and then chimeric sequences in the merged reads were removed. These ASV analyses were conducted with the DADA2 package (version 1.4, https://benjjneb.github.io/dada2) in R (version 3.4.4) [64]. The ASV were assigned by naive Bayesian classifier method with Silva database nr v132 (http://www.arb-silva.de/) [40, 41]. Alpha diversity, beta diversity, principal coordinate analysis (PCoA), and nonmetric multidimensional scaling (NMDS) were visualized with the ggplot2 (version 2.2.1) package in R. Superheat (version 0.1.0). Accession numbers for all sequencing data were recorded in GenBank (Table S6).

For sequencing analysis of rhizosphere populations, growth conditions of tomato plants were as described above. Seedlings of tomato (cv. Heinze 1350) were maintained in a growth chamber under day/night conditions of 16 h light and 8 h dark. Temperature during the light cycle was 25 °C ± 2 and was increased after inoculation of FOL to 28 °C ± 2 to enhance pathogen infection. The dark phase temperature was held at 20 °C ± 2 and humidity was no greater than 85%. Seeds were sown in autoclaved nursery soil and irrigated with 0.1% Hoagland’s solution. Tomato seedlings were grown in sterilized soil for 10 weeks and then 10 ml of l-glutamic acid (5 μg/ml) was applied by drenching 3 times at 3-day intervals between weeks 2 and 3. Strain SP6C4 was cultured in TSB broth containing 20% sucrose and 1% mannitol for sporulation. The harvested spores were washed four times with deionized, distilled H_2_O and the pellet was suspended in 50 ml (OD_600nm_ 0.7 ± 0.05) of 0.1% Hoagland solution containing 0.1% methylcellulose (MC) and inoculated into the soil on week 2 after planting. Seven days later, FOL chlamydospore stock (10^5^ cfu/ml) was inoculated into the soil. The disease index was scored on week 7 using the five-grade scale above. At 10 weeks, the rhizosphere soil of 3 replicate plants was pooled for DNA extraction and sequencing. The seven treatments included an untreated control, FOL alone, l-glutamic acid (Glu) alone, SP6C4 alone, FOL + Glu, FOL + SP6C4, and FOL + Glu + SP6C4. Rhizosphere soil (0.5 g) was added to lysing matrix E and DNA was extracted using a FastDNA Spin Kit (MP Bio). The DNA was suspended in 50 μl of DES buffer and the tubes were stored at – 20 °C for sequencing and *lanM* qPCR. For sequencing, 200 ng of DNA was precipitated with ethanol and the V4 region of 16S rRNA was amplified with primers 515F forward (5′-gtgycagcmgccgcggtaa-3′) and 806R reverse (5′-ggactacnvgggtwtctaat-3′). PCR products were subjected to Illumina MiSeq 250-bp paired-end sequencing at Macrogen (Daejeon, Korea). For correction of 16S rRNA amplicon, the 3′-end in the reads was trimmed of low-quality reads (average quality score of sequence location < 30) and primer sequences. The quality scores were subjected to machine learning according to DADA2 [64] by the ASV method and the learned content was used to calibrate the sequences. The calibrated reads were merged from pair of forward and reverse sequences, chimeras were removed, and clustered as ASVs based on equal nucleotide sequences. For the series of ASV method procedures, DADA2 (version 1.4, https://benjjneb.github.io/dada2) was used. Each ASV was identified by the naive Bayesian classifier method and reference to Silva database version 138 (https://www.arb-silva.de/) using Id taxa classifier [41]. Dominant ASVs were plotted as PCoA and beta diversity bars using ggplot2 in the R package (R, version 3.4.4). Other visualizations were made using superheat (version 0.1.0) for heatmaps, ASV relative abundance was calculated with NOI-seq (version 3.10), and correlations were calculated with Spearman’s method. All sequencing data and GenBank accession numbers were recorded in Table S6.

### Disease incidence of gray mold and blossom blight and qPCR of lanM

Flowers (*Fragariae x ananassa* cv. Meahyang) were collected from a 660 m^2^ greenhouse with 15 plots of 1.5 × 3 m^2^, each with 100 strawberry plants. Each of three treatments (untreated control, l-glutamic acid or l-asparagine at a final concentration of 2%, pH 6.5) in five randomly arranged replicate plots was sprayed for 1 min per plot (Sprayer: HP-2010, Korea, 1.5 L discharge capacity min^−1^) at 2-week intervals during January and February, 2018. Five samples per plot, each with 3 to 5 flowers, were collected into 50-ml Falcon tubes at 2-week intervals from December, 2017 through February, 2018, chilled on ice to preserve microbial communities, and transported to the laboratory for sequence analysis. The incidence of gray mold and blossom blight caused by *Botrytis cinerea* and *Cladosporium* spp., respectively, was expressed as the percentage of infected plants in a greenhouse of 9 plots, each with 100 strawberry plants. Early symptoms of gray mold included brown spots on flower petals and were followed by gray conidia covering flowers and fruits [65]. Blossom blight appeared as gray fungal growth on flower pistils and stamens and as infected, malformed, or misshapen fruits [66]. Differences in disease incidence among an untreated control and treatments with 2% glutamic acid or l-asparagine were analyzed by the paired Kruskal-Wallis test and compared for mean separation with the untreated plots with Tukey’s HSD (*P* = 0.05) [67, 68]. To determine whether the population size of the core microbe *S*. *globisporus* SP6C4 increased in response to the amino acid treatments, microbial DNA from the flower anthosphere was extracted and the SP6C4-specific marker gene *lanM* was quantified by qPCR with F and R primers as described by Kim et al. [39]. qPCR reactions in SYBR Green® TOYOBO master mix included denaturation at 98 °C for 5 min followed by 40 cycles of denaturation at 98 °C for 30 s, annealing at 59 °C for 30 s and elongation at 72 °C for 45 s with a CFX Connect™ Optics Module Real-Time PCR System (Bio-Rad, Hercules, CA, USA).

### Disease suppressiveness in tomato by glutamic acid

For assays of Fusarium wilt disease control, tomato plants (cv. Heinze 1350) were maintained in a plant growth chamber for 4 weeks. Conditions included a 16-h day cycle at 27 ± 2 °C and an 8-h night cycle at 20 ± 2 °C, both at 80% relative humidity. Seed was sterilized in 1.5% NaOCl for 30 min with gentle shaking and washed 3 times with ddH_2_O. The seeds were germinated on damp cotton in a Petri dish (9-cm, diam.) for 3 days at 4 °C and then transferred to plastic pots (10-cm, diam.) with autoclaved nursery soil. After 7 days’ germination, 10 ml of l-glutamic acid (5 μg/ml) was added by drenching twice in weeks 1 and 3, 10^6^ cfu/ml of SP6C4 bacterial stock (10 ml, with 0.1% MC) was added in week 4, and 10^5^ cfu/ml of *Fusarium oxysporum* f. sp. *lycopersici* (FOL) chlamydospores (10 ml) were drenched into the soil in week 6. Images of stem and leaf growth were captured 2 weeks later. Shoot length and shoot fresh weight were scored at 10 weeks and disease index was determined 0 d.p.i, 5 d.p.i, and 15 d.p.i with 6 levels: (0, no symptoms; 1, slight yellowing of the lower leaves; 2, moderate yellowing of the entire plant; 3, wilted plant; 4, plants severely stunted or browning; 5, plants dead). All treatments had 3 biological replications and the results was calculated by Kruskal-Wallis test and Tukey HSD tests in R (version 3.4.4.) [67, 68].

### Effect on disease suppression of antibiotic and amino acid treatments to reshape the core microbiota

Strawberry seedlings (cv. Meahyang) were stored at – 2 °C for 1 month for vernalization and then transferred to plastic pots (10 cm diameter). Twelve days after planting, each plant had 5–7 flowers. Then, l-glutamic acid (5 μg/ml) and the antibiotics erythromycin and clindamycin (10 μg/ml each, to inhibit *Streptomyces*, Research Products International, Mt. Prospect, IL, USA) [69–72] were applied with a sprayer. Three days later, freshly grown conidia of *B*. *cinerea* were collected with a cheese cloth filter and sprayed at 10^5^ cfu/ml on the flowers. The seven treatments of 5 plants each included an untreated control, pathogen only (*B*. *cinerea*), l-glutamic acid only, antibiotics only, Glu + pathogen, antibiotics + pathogen, and Glu + antibiotics + pathogen. All plants were maintained in a growth chamber with a daytime temperature of 25 °C ± 3; a nighttime temperature of 15 °C ± 3; and relative humidity of 85%. Two weeks later, disease incidence was scored on 30 flowers (10 independent replicates) and analyzed by the Kruskal-Wallis test and Tukey HSD tests in R (version 3.4.4.) [67, 68].

Seedlings of tomato (cv. Heinze 1350) were maintained in a growth chamber under day/night conditions of 16 h light and 8 h dark. Temperature during the light cycle was 25 °C ± 2 and was increased after inoculation of FOL to 28 °C ± 2 to enhance pathogen infection. The dark phase temperature was held at 20 °C ± 2 and humidity was no greater than 85%. Seeds were sown in autoclaved nursery soil and irrigated with 0.1% Hoagland’s solution. After 12 days, the seedlings were treated with a 10 ml mixture of l-glutamic acid (5 μg/ml) and antibiotics (erythromycin and clindamycin, each at 10 μg/ml). FOL inoculation was performed with a chlamydospore stock solution (10^5^ cfu/ml) at 15 days, and each treatment have 10 biological replications. At the final of sampling time (week 6), the wilt disease index was determined for 5 independent plants as 6 levels: 0, no symptoms; 1, slight yellowing of the lower leaves; 2, moderate yellowing of the entire plant; 3, wilted plant; 4, plants severely stunted or browning; 5, plants dead. Disease index data was calculated by Kruskal-Wallis test and Tukey HSD test in R (version 3.4.4.) [67, 68].

### Quantitative PCR analyses of lanM (grisin) gene

Quantitative PCR analysis of the of *lanM* grisin gene, a specific marker for strain SP6C4, was used to determine the population size of the bacteria in disease suppression assays from samples of strawberry flowers or the tomato rhizosphere. One gram of flower or rhizosphere material (*n* = 3; 5 technical replications) was collected at weeks 2 or 6, respectively and ground with liquid nitrogen. The flower powder (0.5 g) or the tomato rhizosphere (0.2 g) was added to a 2-ml lysing matrix E Fast DNA™ Spin Kit for Soil DNA extraction (MP Biomedicals). The tube, with 978 μl of sodium phosphate buffer and 122 μl of MT buffer, was lysed with a FastPrep®-24 at 6.5 m/sec for 45 s. The solution was cleared after lysis by centrifugation (10 min, 12,470×*g*) and the supernatant was transferred to a 2.0-ml E-tube with 250 μl of PBS buffer. Next, a DNA™ Spin Kit for Soil DNA extraction (MP Biomedicals) was used according to the manufacturer’s instructions with 978 μl of sodium phosphate buffer and 122 μl of MT buffer, followed by qPCR of *lanM* with specific primers F: 5′ gtacggatctgcaccacga 3′ and R: 5′ aacagggtctccacatcgac 3′. PCR condition included initial denaturation 98 °C for 5 min; 40 cycles of denaturation at 98 °C for 30 s; annealing at 59 °C for 30 s, and elongation at 72 °C for 45 sec with a CFX Connect™ Optics Module Real-Time PCR System (Bio-Rad, Hercules, CA, USA) [39]. All data were analyzed by the Kruskal-Wallis test and compared for mean separation with the untreated plots by Tukey’s HSD (*P* = 0.05) [67, 68].

### Statistical analyses

In all case, normality was confirmed with the Shapiro-Wilk test and Levene’s test for homogeneity of variance prior to further analysis**.** For *Illumina sequencing,* data visualization of beta diversity was carried out with PCoA and NMDS plots and statistical analysis was by PERMANOVA. Other data were analyzed by Kruskal-Wallis (*P* < 0.05) and comparisons were used to demonstrate differences among mean values with Tukey’s HSD (*P < 0.05, **P < 0.01, ***P < 0.001) [67, 68]. All graphs were visualized by ggplots version 3.0.1 and ggplot2 version 2.1.0 in the R software package.

## Supplementary Information

**Additional file 1: Figure S1.** Chemical constituents of strawberry flower exudate. **Figure S2.** Biolog phenotype array for nitrogen utilization and bacterial growth. **Figure S3.** Dynamics of strawberry flower microbial communities as influenced by amino acids. **Figure S4.** Metacoder analysis of the microbial composition of strawberry flowers among treatments (untreated, 2% L-glutamic acid, 2% L-asparagine). **Figure S5.** Gray mold and blossom blight disease incidence. **Figure S6.** Comparison of the tomato rhizosphere microbiome at the family level by PCoA. **Figure S7.** Experimental design in the strawberry greenhouse. **Figure S8.** Suppression of Fusarium wilt disease of tomato by strain SP6C4 with or without L-glutamic acid.

**Additional file 2: Table S1.** Climate data of the strawberry greenhouse from November 2013 to March 2014. **Table S2.** Optical density of PM1 plate for carbon sources (96-well format). **Table S3.** Optical density of PM3B plate for nitrogen sources (96-well format). **Table S4.** Number of sequencing read counts of strawberry flower samples. **Table S5.** Number of sequencing read counts of tomato rhizosphere samples. **Table S6.** GenBank accession numbers for strawberry flower sample pyrosequencing.

**Additional file 3.** Strawberry anthosphere metagenome OTU data.

**Additional file 4.** Tomato rhizosphere metagenome OTU data.

## Data Availability

Sequencing data for l-glutamic acid-treated flower samples have been deposited in GenBank under SAR accession number SRR11355399 [https://www.ncbi.nlm.nih.gov/sra/SRR11355399] and all other GenBank data in Table S6. All data are available in the manuscript the supplementary materials and analyses of microbial community composition were carried out with R program (version 3.4.4). The source code of R for data analyses is available on GitHub at https://github.com/ekfks0125/2020_Kim.git.

## Abbreviations

SP6C4: *Streptomyces globisporus* SP6C4
PM: Phenotype microarray
DADA2: Divisive amplicon denoising algorithm 2
ASV: Amplicon sequencing variants
PCoA: Principal coordinate analysis
qPCR: Quantitative polymerase chain reaction
BC: *Botrytis cinerea*
FOL: *Fusarium oxysporum* f. sp. *lycopersici*
RA: Relative abundance
DI: Disease incidence
NMDA: Non-metric multidimensional scaling
AA: Amino acid
OA: Organic acid
ECF: Ethyl chloroformate
MTBSTFA: Methyl-*N*-(*tert*-butyldimethylsilyl) trifluoroacetamide
HPLC: High-performance liquid chromatography
PVDF: Polyvinylidene difluoride
GC–MS: Gas chromatography-mass spectrometry
SIM: Selected ion monitoring
EOC: Ethoxycarbonylation
MC: Methylcellulose
GC–SIM–MS: *Gas chromatography–*mass spectrometry/selective ion monitoring
Glu: l-glutamic acid
