# Supplementary material for: Glutamic acid reshapes the plant microbiota to protect plants against pathogens
Source: Microbiome. 2021 Dec 20;9:244. doi: 10.1186/s40168-021-01186-8 (PMC8691028; doi:10.1186/s40168-021-01186-8)
Supplement: Supplementary file 2 — Additional file 1: Figure S1. Chemical constituents of strawberry flower exudate. Figure S2. Biolog phenotype array for nitrogen utilization and bacterial growth. Figure S3. Dynamics of strawberry flower microbial communities as influenced by amino acids. Figure S4. Metacoder analysis of the microbial composition of strawberry flowers among treatments (untreated, 2% L-glutamic acid, 2% L-asparagine). Figure S5. Gray mold and blossom blight disease incidence. Figure S6. Comparison of the tomato rhizosphere microbiome at the family level by PCoA. Figure S7. Experimental design in the strawberry greenhouse. Figure S8. Suppression of Fusarium wilt disease of tomato by strain SP6C4 with or without L-glutamic acid. [file 40168_2021_1186_MOESM2_ESM.docx]

**Supplementary information**

***for***

**Glutamic acid reshapes the plant microbiota to protect plants against pathogens**

Da-Ran Kim^1^, Chang-Wook Jeon^2^, Gyeongjun Cho^2^, Linda S. Thomashow^3^, David M. Weller^3^, Man-Jeong Paik^4^, Yong Bok Lee^2^, and Youn-Sig Kwak^1,2,5,^*

**Additional file 1:**

**Figure S1.** Chemical constituents of strawberry flower exudate.

**Figure S2.** Biolog phenotype array for nitrogen utilization and bacterial growth.

**Figure S3.** Dynamics of strawberry flower microbial communities as influenced by amino acids.

**Figure S4.** Metacoder analysis of the microbial composition of strawberry flowers among treatments (untreated, 2% L-glutamic acid, 2% L-asparagine).

**Figure S5.** Gray mold and blossom blight disease incidence.

**Figure S6.** Comparison of the tomato rhizosphere microbiome at the family level by PCoA.

**Figure S7.** Experimental design in the strawberry greenhouse.

**Figure S8.** Suppression of Fusarium wilt disease of tomato by strain SP6C4 with or without L-glutamic acid.

**
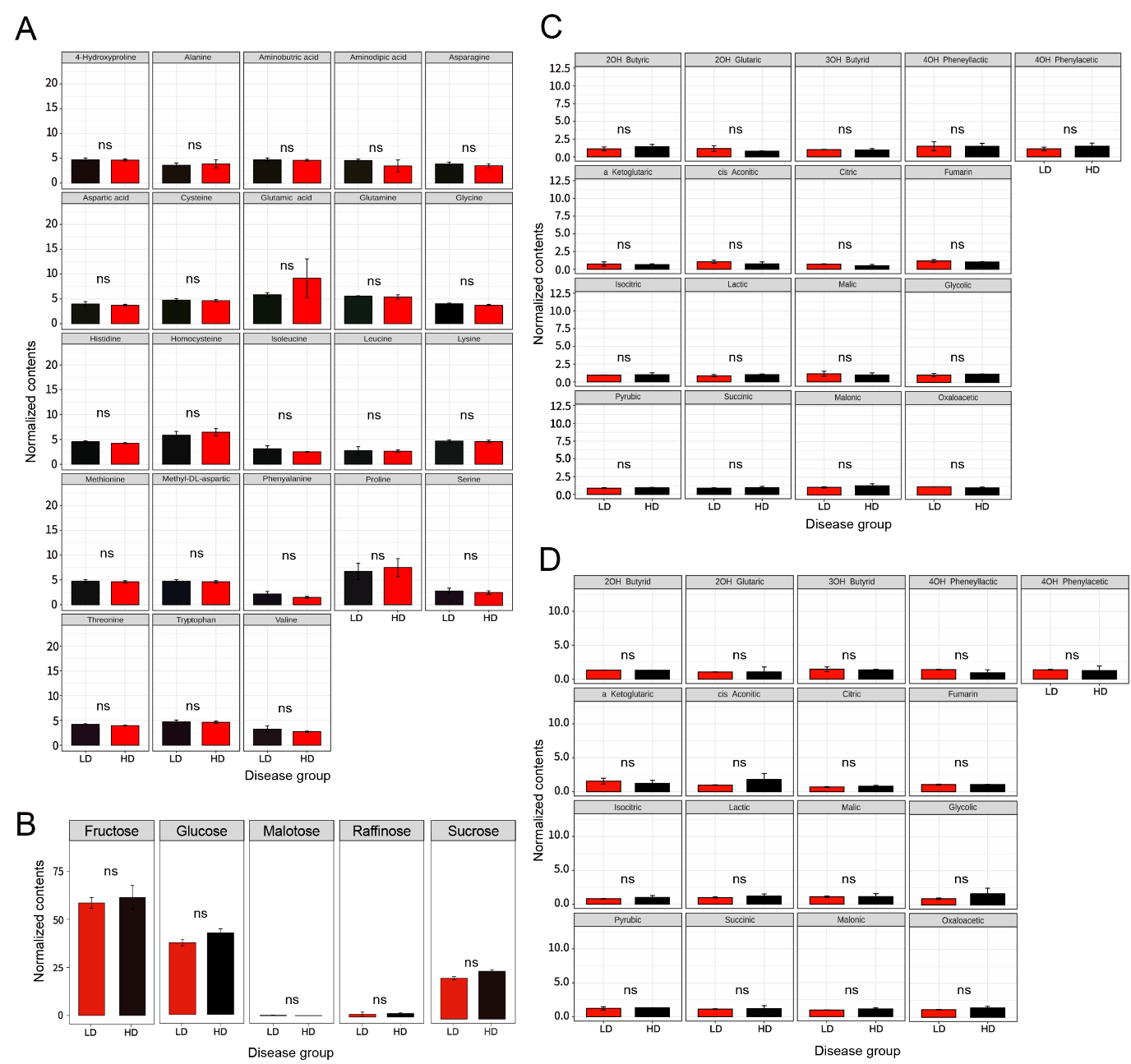
**

**Figure S1: Chemical constituents of strawberry flower exudate.** **A** Content of 23 types of amino acids in the strawberry ovary (*n* = 3, 8 independent experiments). Normalized concentrations of amino acids were tested by independent *t*-test (*p* value < 0.05). **B** sugar contents (*n* = 3, 8 independent experiments). **C** Organic acid contents of strawberry flower petals (*n* = 3, 8 independent experiments) and **D** ovary (*n* = 3, 8 independent experiments).


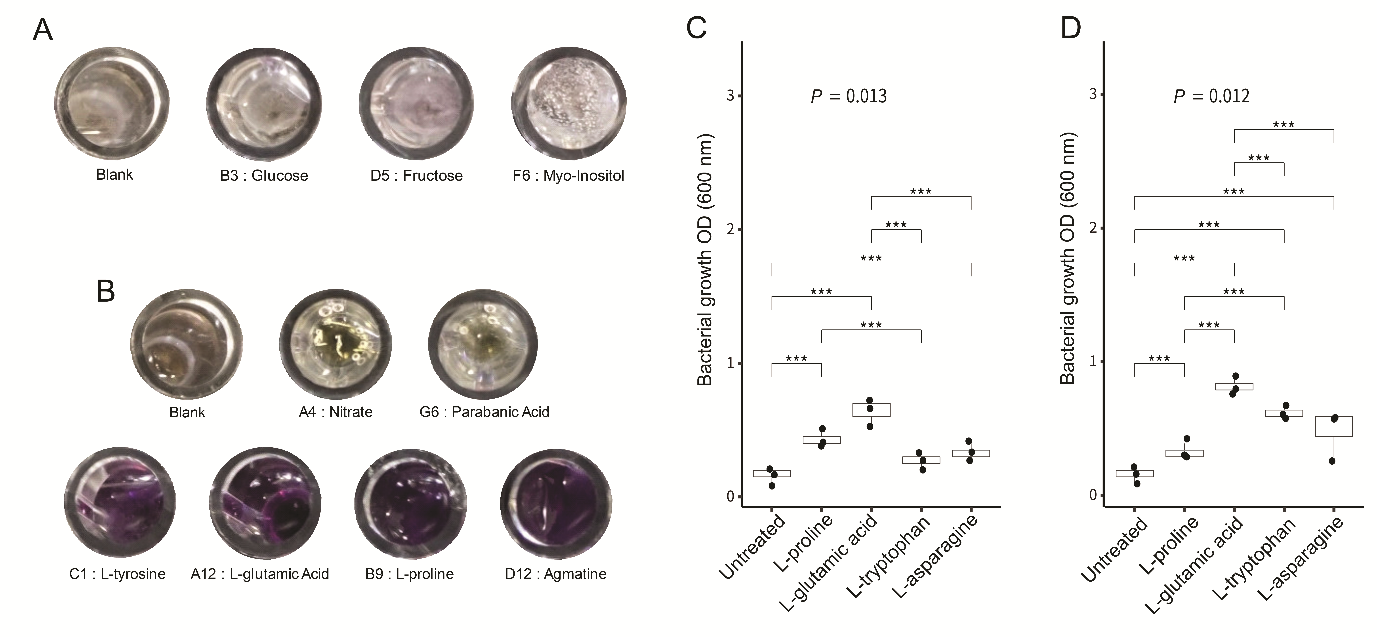


**Figure S2: Biolog phenotype array for nitrogen utilization and bacterial growth.** Biolog plates (*n* = 3, 3 independent experiments) were inoculated with strain SP6C4 (OD_600nm_ 0.2) in 0.2% carrageenan and incubated at 28°C for 3 days. Redox dye mix MA was added when the second or the fourth wells in a row turned yellow and a color change to dark purple was observed at 37°C. The color was assayed at 600 nm. **A** PM1 plate for carbon sources, all intensity data presented in Table S2. **B** PM3B plate for nitrogen sources, all intensity data presented in Table S3. **C** Growth of SP6C4 in basal medium amended or not with 0.02% amino acids. **D** Growth of strain SP6C4 with 0.2% amino acids. Box plot represent Kruskal-Wallis rank-sum test and stars indicate statistically significant differences among treatments in Tukey's HSD (**P* < 0.05, ***P* < 0.01, ****P* < 0.001).


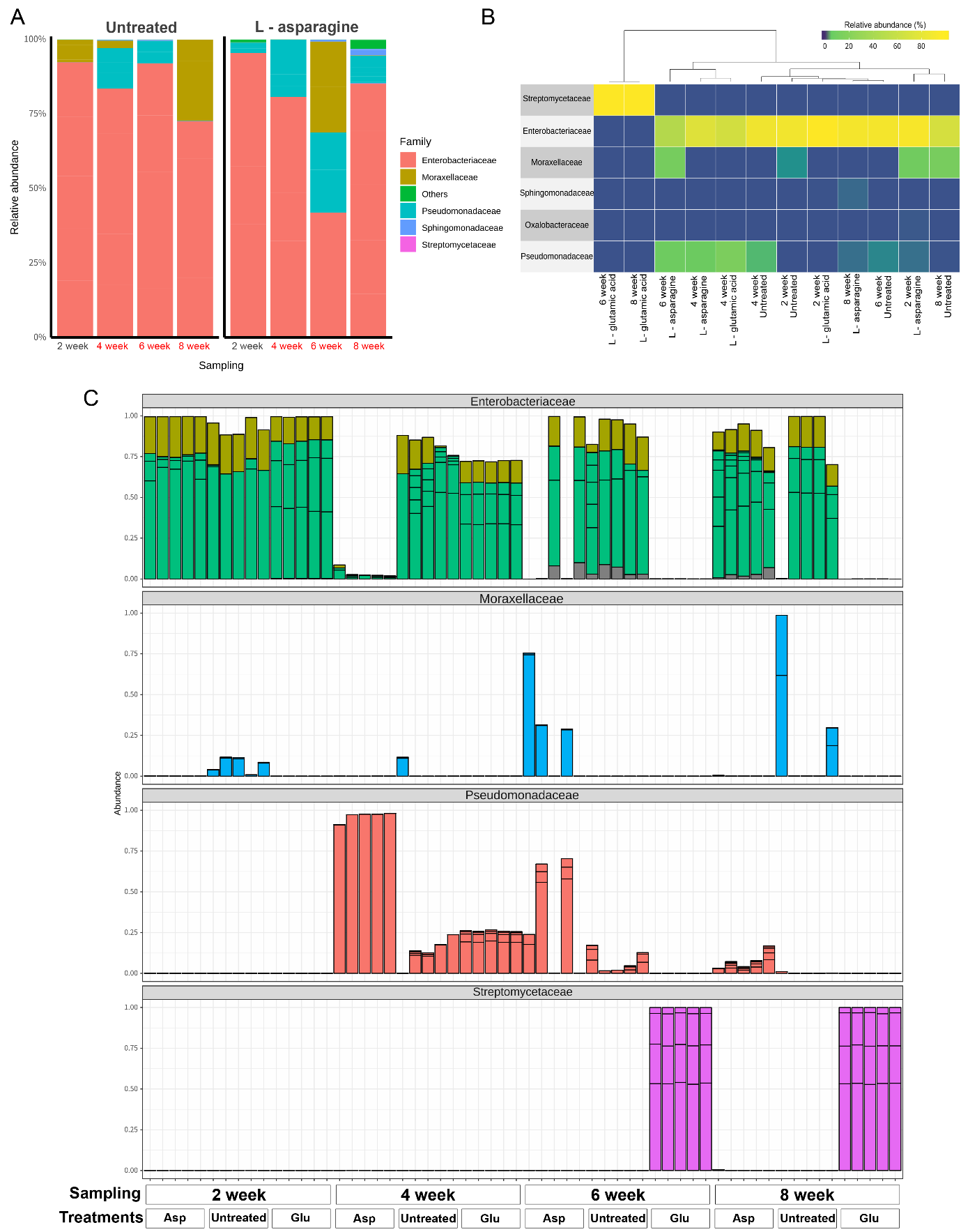


**Figure S3: Dynamics of strawberry flower microbial communities as influenced by amino acids**. Samples were collected from December 2017 to February 2018 and amino acids were sprayed from Jan, 2018 to Feb, 2018 (weeks 4, 6 and 8). Sequencing of the microbes associated with strawberry flowers (*n* = 5, 12 independent experiments). Taxonomic assignment was conducted at the family level in the Silva database (http://www.arb-silva.de/) with a similarity cutoff of 97% confidence. **A** Taxonomic profiles showing the microbial diversity with bar plots. Bar plots displaying the relative abundance at the family level. **B** Similarity of microbial abundance by hierarchical clustering of the variable region of 16S rRNA with a beta diversity tree (Minkowski distance method). Heatmap color (purple to yellow) displays low to high abundance of each ASV. **C** Taxonomic profiles showing the microbial diversity with bar plots. Bar plots displayed the relative abundance at the family level and taxonomic assignment conducted with the Silva database (http://www.arb-silva.de/).


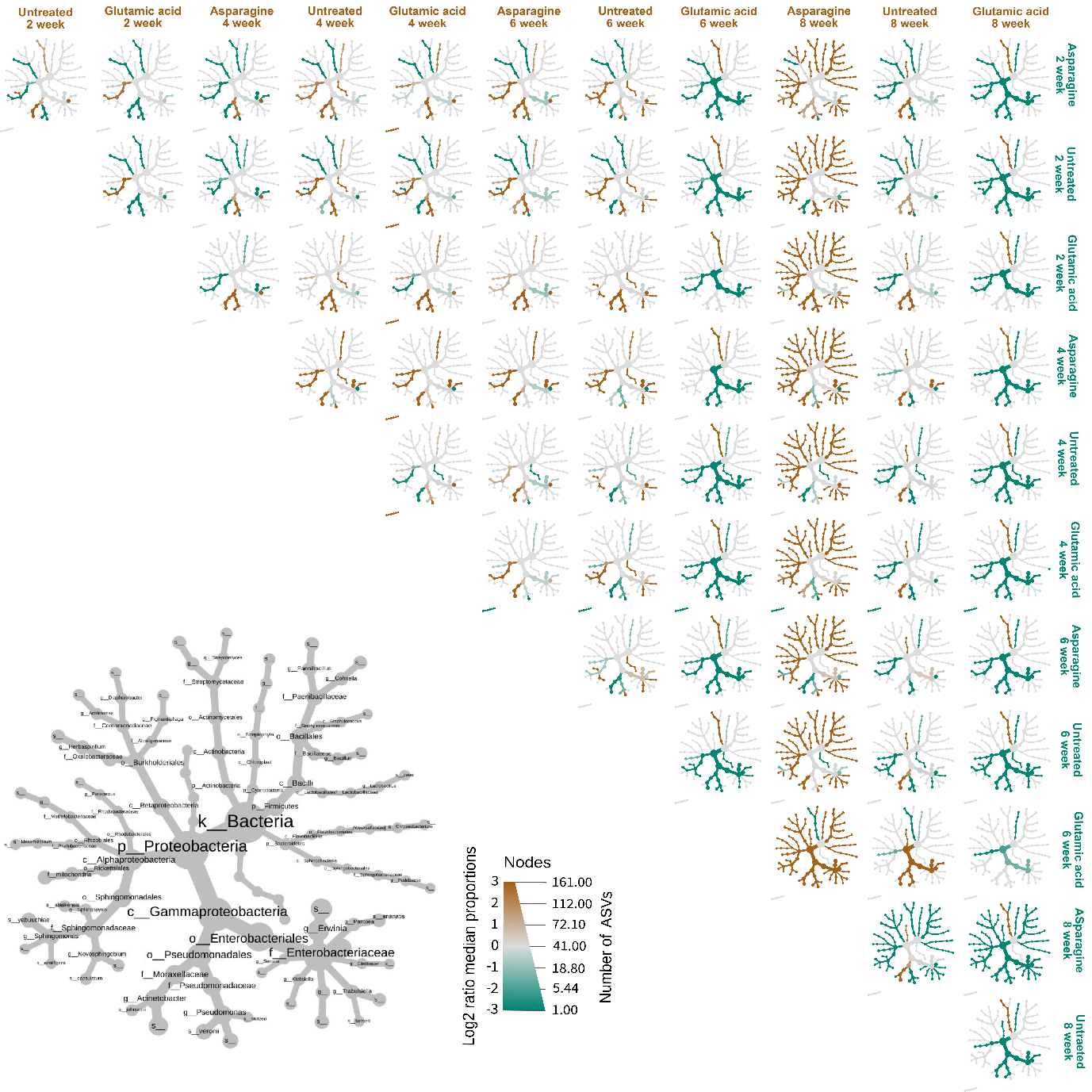


**Figure S4: Metacoder analysis of the microbial composition of strawberry flowers among treatments (untreated, 2% L-glutamic acid, 2% L-asparagine).** Phylotrees showing diversity of microbial ASVs in L-glutamic acid-treated flowers contrasted with diversity in untreated flowers or flowers sprayed with L- asparagine (Metacoder v 0.3.0.1). Size of the nodes refers of the relative abundance and color represents a significant change in relative abundance. Trees were generated by using maximum likelihood to generate a phyloseq object in Phyloseq (version 3.10). The node color indicates significant changes in relative abundance (Log2 ratio).


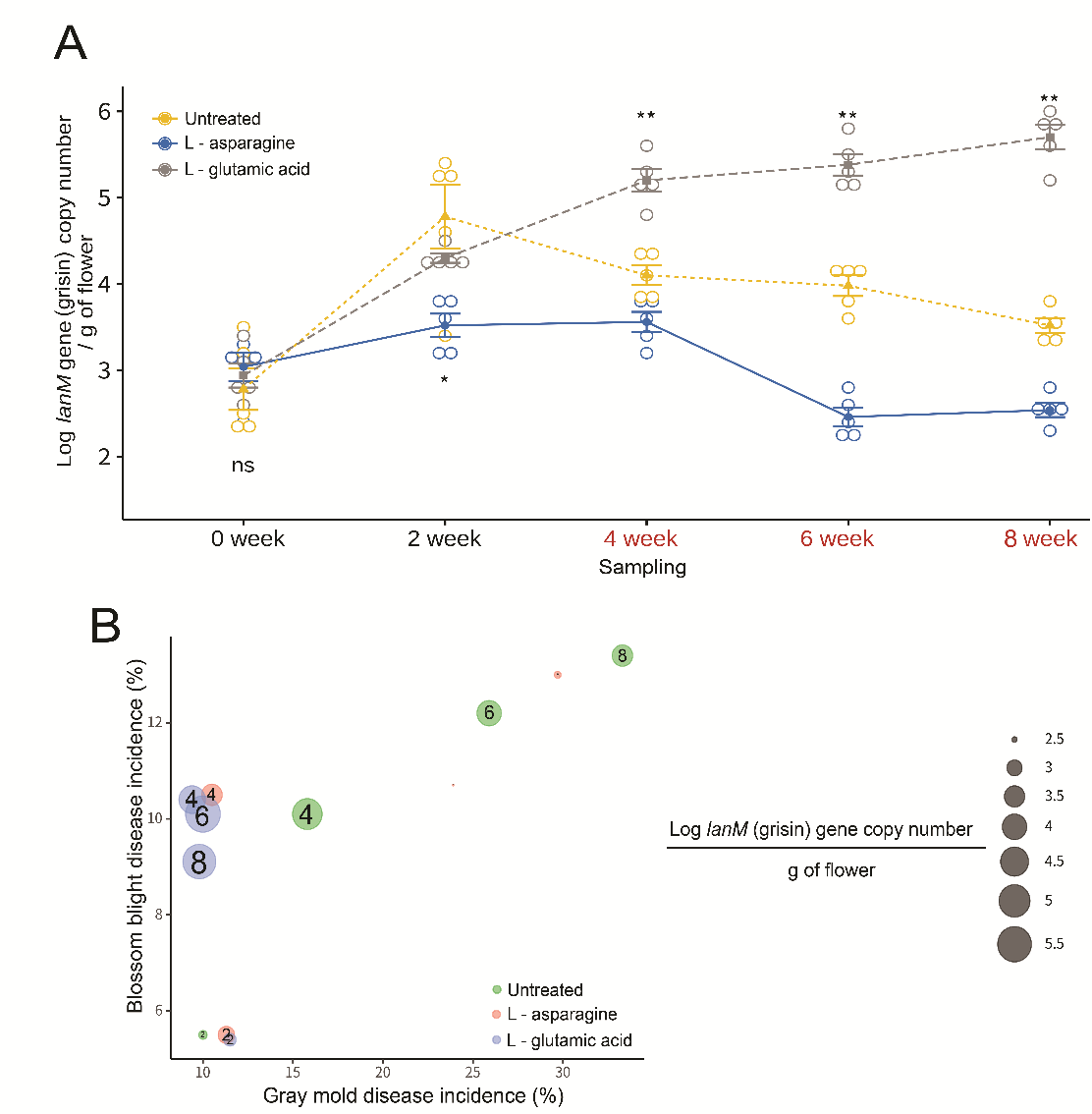


**Figure S5: Gray mold and blossom blight disease incidence**. Plants in the greenhouse (8 M wide X 82.5 M length; 660 m^2^) were arranged in 7 longitudinal rows, 3 of which were untreated; 2% L–asparagine and 2% L–glutamic acid were applied by sprayer (HP-2010, Korea, 1.5 L discharge capacity/min) at intervals of 2 weeks. Mean disease incidence of 5 independent plots ± SE; red letters in the legend indicate when amino acids were sprayed. **A** Detection of *S. globisporus* SP6C4 with the strain specific *lanM* gene by qPCR. Strawberry flower samples were collected into 50 mL Falcon tubes, each with 3 flowers. DNA was extracted by with a Fast DNA^TM^ Spin Kit for Soil DNA extraction (*n* = 5, 15 independent experiments) and qPCR was performed with the CFX connect real-time PCR detection system (Bioneer, Korea). **B** Circle size indicates *S. globisporus* SP6C4 population density and numbers in circles indicate sampling time. All data were tested by Kruskal-Wallis rank-sum test with Tukey's HSD, Bars indicate the standard error and stars represent significance at **P* < 0.05, ***P* < 0.01, ****P* < 0.001.

**
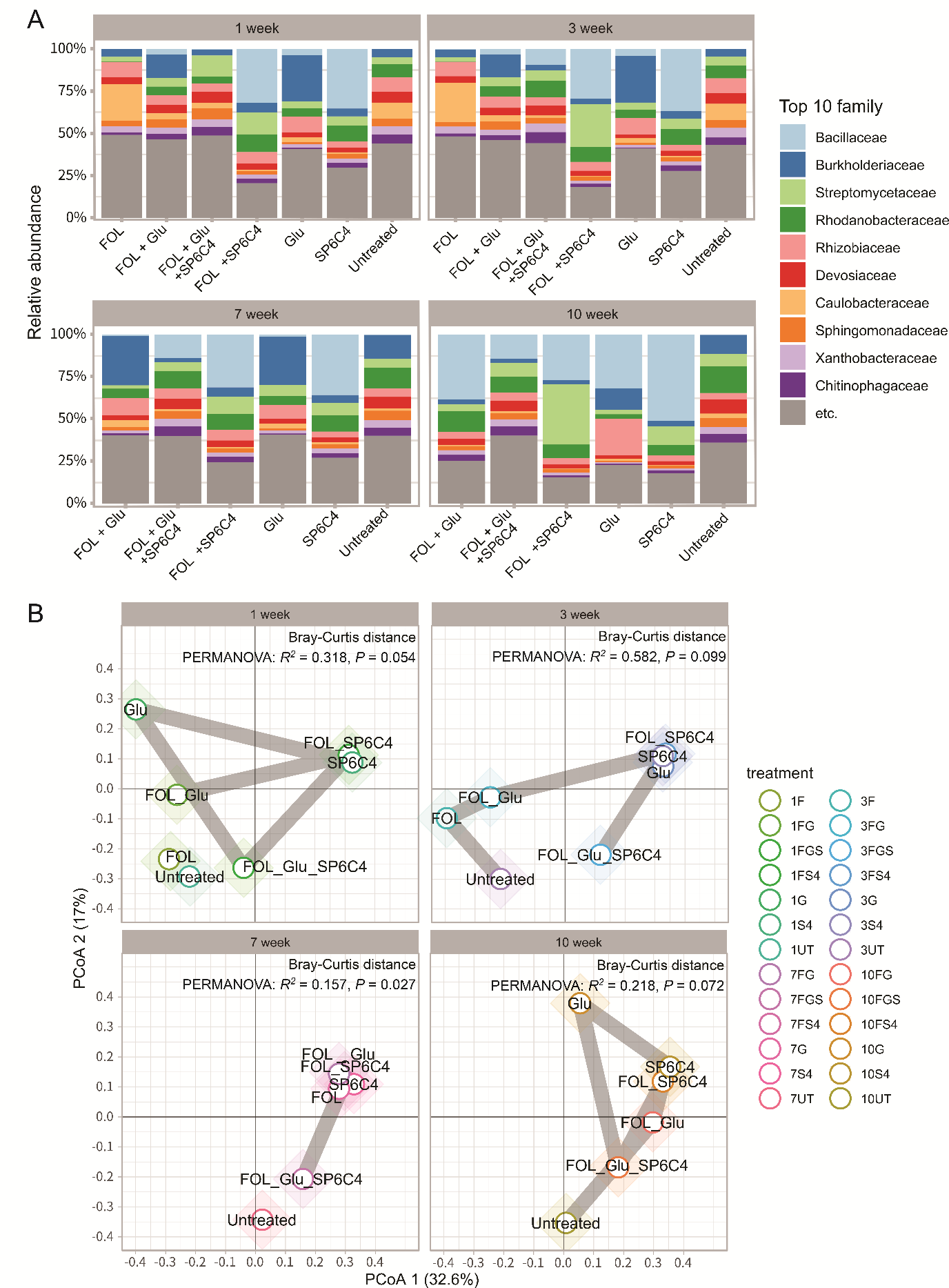
 Figure S6:** **Comparison of the tomato rhizosphere microbiome at the family level by PCoA.** **A** Sequencing analysis with the DADA2 package and taxonomic assignment were performed with Silva database v138 on Id taxa. Top 10 families are shown in the bar plots. **B** Illumina sequencing data of the tomato rhizosphere as presented by PCoA with weekly samples calculated by PERMANOVA.


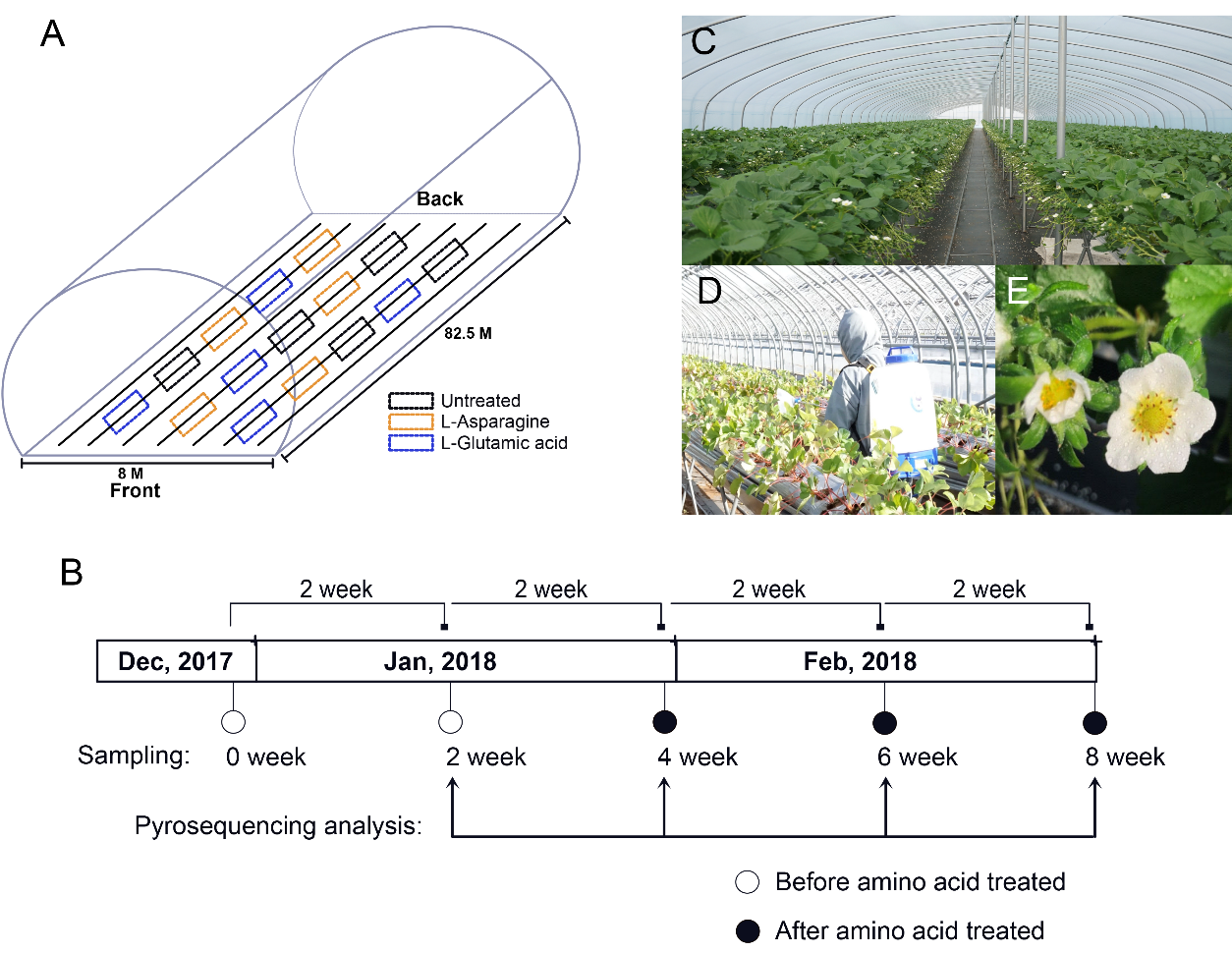


**Figure S7: Experimental design in the strawberry greenhouse. A** Layout of the 660 m^2^ greenhouse with 15 plots of 1.5 X 3 m^2^, consisting of five replicates each of three treatments (untreated control L-glutamic acid, L-asparagine) in a randomized block design. Each plot contained 100 strawberry plants. **B** L–glutamic acid (2%) and L–asparagine (2%) were applied at weeks 4, 6 and 8 by sprayer (HP-2010, Korea, 1.5 L discharge capacity/min) and flowers were sampled for sequencing. **C** Strawberry greenhouse; **D, E** Flowers were sprayed, and samples were collected into a 50-mL Falcon tube (*n* = 5).

**
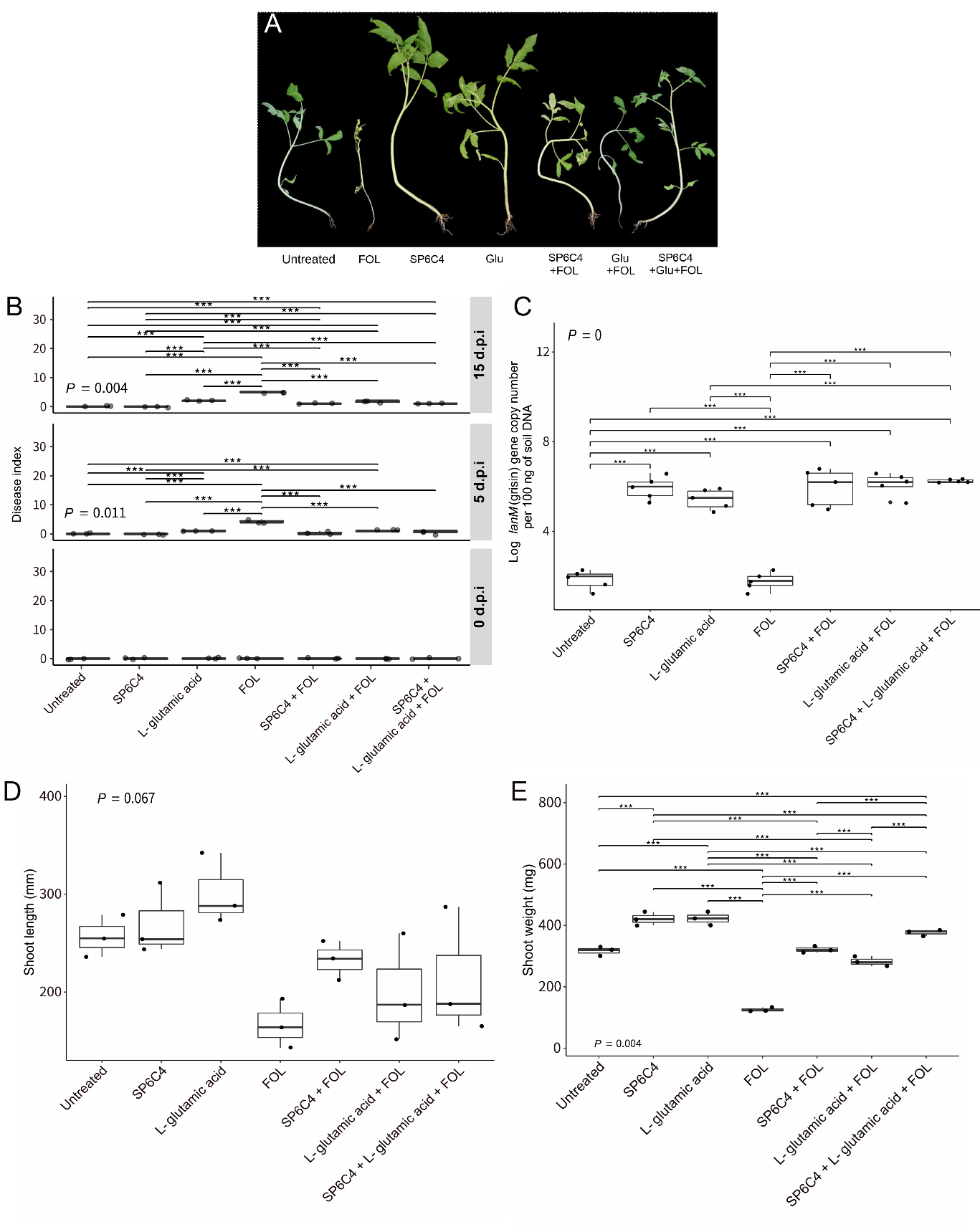
Figure S8: Suppression of Fusarium wilt disease of tomato by strain SP6C4 with or without L-glutamic acid.** Plants were maintained in a growth chamber (light: 16 h, 28°C, RH 80%; dark: 8 h, 25°C, RH 80%). **A** seedlings at week 2. **B** Fusarium wilt disease progress over time, disease index was scored by using a scale of five grades. The data are presented as box plots, *n* = 3. **C** *lanM* gene copy number determined by qPCR of rhizosphere soil DNA. Soil (0.5 g) was collected from the plant rhizosphere (three biological replicates), DNA was isolated by using a FastDNA^TM^ Spin kit (MP biomedicals, USA). **D, E** Phenotype of tomato seedlings at 4 weeks. Data were calculated by the Kruskal-Wallis rank-sum test and significant differences were analyzed by Tukey's HSD of all data; **P* < 0.05, ***P* < 0.01, ****P* < 0.001.
