# Supplementary material for: Glutamic acid reshapes the plant microbiota to protect plants against pathogens
Source: Microbiome. 2021 Dec 20;9:244. doi: 10.1186/s40168-021-01186-8 (PMC8691028; doi:10.1186/s40168-021-01186-8)
Supplement: Supplementary file 3 — Additional file 2: Table S1. Climate data of the strawberry greenhouse from November 2013 to March 2014. Table S2. Optical density of PM1 plate for carbon sources (96-well format). Table S3. Optical density of PM3B plate for nitrogen sources (96-well format). Table S4. Number of sequencing read counts of strawberry flower samples. Table S5. Number of sequencing read counts of tomato rhizosphere samples. Table S6. GenBank accession numbers for strawberry flower sample pyrosequencing. [file 40168_2021_1186_MOESM3_ESM.docx]

**Supplementary information**

***for***

**Glutamic acid reshapes the plant microbiota to protect plants against pathogens**

Da-Ran Kim^1^, Chang-Wook Jeon^2^, Gyeongjun Cho^2^, Linda S. Thomashow^3^, David M. Weller^3^, Man-Jeong Paik^4^, Yong Bok Lee^2^, and Youn-Sig Kwak^1,2,5,^*

**Additional file 2:**

**Table S1** Climate data of the strawberry greenhouse from November 2013 to March 2014

**Table S2** Optical density of PM1 plate for carbon sources (96-well format)

**Table S3** Optical density of PM3B plate for nitrogen sources (96-well format)

**Table S4** Number of sequencing read counts of strawberry flower samples

**Table S5** Number of sequencing read counts of tomato rhizosphere samples

**Table S6** GenBank accession numbers for strawberry flower sample pyrosequencing

**Table S1:** Climate data of strawberry green house for November 2013 to March 2014

| Sampling | Air temperature | Soil temperature | Humidity | CO_2_ | Disease incidence | EC | pH |
| --- | --- | --- | --- | --- | --- | --- | --- |
| 2 week | 12.78 | 17.41 | 71.08 | 494.01 | 0.5 | 6.3 | 7 |
| 4 week | 13.68 | 16.49 | 71.76 | 421.44 | 0.33 | 6.3 | 7 |
| 6 week | 11.15 | 14.58 | 79.89 | 408.47 | 1 | 6.4 | 7 |
| 8 week | 11.50 | 13.99 | 66.63 | 331.43 | 1 | 6.3 | 7 |
| 10 week | 12.27 | 15.02 | 64.49 | 358.32 | 1 | 6.4 | 7 |
| 12 week | 11.29 | 14.48 | 78.00 | 279.25 | 1.21 | 6.2 | 7 |
| 14 week | 12.40 | 16.44 | 65.84 | 358.17 | 2.83 | 6.1 | 7 |
| 16 week | 13.26 | 15.15 | 72.05 | 475.00 | 6 | 6.3 | 7 |
| 18 week | 16.65 | 18.27 | 91.23 | 564.03 | 13.17 | 6.4 | 7 |
| 20 week | 17.01 | 18.75 | 64.96 | 478.20 | 32 | 6.3 | 7 |
| 22 week | 18.65 | 20.27 | 66.79 | 484.74 | 30.83 | 6 | 7 |
| 24 week | 17.66 | 17.58 | 91.73 | 446.05 | 28 | 6 | 7 |

**Table S2:** Optical density of PM1 plate for carbon sources (96-well format)

|  | 1 | 2 | 3 | 4 | 5 | 6 | 7 | 8 | 9 | 10 | 11 | 12 |
| --- | --- | --- | --- | --- | --- | --- | --- | --- | --- | --- | --- | --- |
| A | Blank | L-Arabinose | N-Acetyl-D-  Glucosamine | D-Saccharic Acid | Succinic Acid | D-Galactose | L-Aspartic Acid | L-Proline | D-Alanine | D-Trehalose | D-Mannose | Dulcitol |
|  | 0.16 a | 0.13 a | 0.119 a | 0.15 a | 0.18 a | 0.16 a | 0.12 a | 0.35 a | 0.11 a | 0.19 a | 0.17 a | 0.11 a |
| B | D-Serine | D-Sorbitol | Glycerol | L-Fucose | D-Glucuronic Acid | D-Gluconic Acid | D,L- α -GlycerolPhosphate | D-Xylose | L-Lactic Acid | Formic Acid | D-Mannitol | L-Glutamic Acid |
|  | 0.16 a | 0.23 a | 0.23 a | 0.13 a | 0.11 a | 0.1 a | 0.11 a | 0.26 a | 0.16 a | 0.17 a | 0.22 a | 0.39 b |
| C | D-Glucose -6 - Phosphate | D-Galactonic Acid - γ -Lactone | D,L-Malic Acid | D-Ribose | Tween 20 | L-Rhamnose | D-Fructose | Acetic Acid | α -D-Glucose | Maltose | D-Melibiose | Thymidine |
|  | 0.264 a | 0.11 a | 0.22 a | 0.15 a | 0.15 a | 0.1 a | 0.1 a | 0.12 a | 0.2 a | 0.19 a | 0.198 a | 0.19 a |
| D | L-Asparagine | D-Aspartic Acid | D-Glucosaminic Acid | 1,2 -Propanediol | Tween 40 | α -Keto -Glutaric Acid | α -Keto -Butyric Acid | α -Methyl-DGalactoside | α -D-Lactose | Lactulose | Sucrose | Uridine |
|  | 0.32 b | 0.13 a | 0.2 a | 0.27 a | 0.15 a | 0.15 a | 0.16 a | 0.23 a | 0.28 a | 0.12 a | 0.15 a | 0.1 a |
| E | L-Glutamine | M-Tartaric Acid | D-Glucose -1 - Phosphate | D-Fructose -6 - Phosphate | Tween 80 | α -Hydroxy Glutaric Acid- γ - Lactone | α -Hydroxy Butyric Acid | ẞ -Methyl-DGlucoside | Adonitol | Maltotriose | 2-Deoxy Adenosine | Adenosine |
|  | 0.11 a | 0.19 a | 0.28 a | 0.23 a | 0.19 a | 0.12 a | 0.21 a | 0.11 a | 0.22 a | 0.12 a | 0.19 a | 0.26 a |
| F | Glycyl-L - Aspartic Acid | Citric Acid | M-Inositol | D-Threonine | Fumaric Acid | Bromo Succinic Acid | Propionic Acid | Mucic Acid | Glycolic Acid | Glyoxylic Acid | D-Cellobiose | Inosine |
|  | 0.12 a | 0.16 a | 0.11 a | 0.15 a | 0.14 a | 0.23 a | 0.26 a | 0.1 a | 0.12 a | 0.23 a | 0.26 a | 0.17 a |
| G | Glycyl-L - Glutamic Acid | Tricarballylic Acid | L-Serine | L-Threonine | L-Alanine | L-AlanylGlycine | Acetoacetic Acid | N-Acetyl- ẞ -DMannosamine | Mono Methyl Succinate | Methyl Pyruvate | D-Malic Acid | L-Malic Acid |
|  | 0.18 a | 0.19 a | 0.26 a | 0.1 a | 0.29 a | 0.23 a | 0.19 a | 0.19 a | 0.14 a | 0.19 a | 0.17 a | 0.18 a |
| H | Glycyl-L - Proline | p-Hydroxy Phenyl Acetic Acid | m-Hydroxy Phenyl Acetic Acid | Tyramine | D-Psicose | L-Lyxose | Glucuronamide | Pyruvic Acid | L-Galactonic Acid - γ -Lactone | D-Galacturonic Acid | Phenylethylamine | 2-Aminoethanol |
|  | 0.2 a | 0.33 a | 0.27 a | 0.21 a | 0.1 a | 0.24 a | 0.14 a | 0.17 a | 0.11 a | 0.13 a | 0.12 a | 0.1 a |

Intensity of color change of the plate was monitored at OD_590_ nm

**Table S3:** Optical density of PM3B plate for nitrogen sources (96-well format)

|  | 1 | 2 | 3 | 4 | 5 | 6 | 7 | 8 | 9 | 10 | 11 | 12 |
| --- | --- | --- | --- | --- | --- | --- | --- | --- | --- | --- | --- | --- |
| A | Blank | Ammonia | Nitrite | Nitrate | Urea | Biuret | L-Alanine | L-Arginine | L-Asparagine | L-Aspartic Acid | L-Cysteine | L-Glutamic Acid |
|  | 0.198 c | 0.235 c | 0.182 c | 0.21 c | 0.235 c | 0.214 c | 0.262 c | 0.273 c | 0.259 c | 0.446 a | 0.402 a | 0.458 a |
| B | L-Glutamine | Glycine | L-Histidine | L-Isoleucine | L-Leucine | L-Lysine | L-Methionine | L-Phenylalanine | L-Proline | L-Serine | L-Threonine | L-Tryptophan |
|  | 0.261 c | 0.27 b | 0.39 b | 0.302 b | 0.273 b | 0.307 b | 0.212 c | 0.254 c | 0.407 a | 0.262 c | 0.249 c | 0.261 c |
| C | L-Tyrosine | L-Valine | D-Alanine | D-Asparagine | D-Aspartic Acid | D-Glutamic Acid | D-Lysine | D-Serine | D-Valine | L-Citrulline | L-Homoserine | L-Ornithine |
|  | 0.463 a | 0.212 c | 0.172 c | 0.12 c | 0.111 c | 0.077 d | 0.149 c | 0.176 c | 0.159 c | 0.22 c | 0.15 c | 0.171 c |
| D | N-Acetyl-D,L - Glutamic Acid | N-Phthaloyl-L-Glutamic Acid | L-Pyroglutamic Acid | Hydroxylamine | Methylamine | N-Amylamine | N-Butylamine | Ethylamine | Ethanolamine | Ethylenediamine | Putrescine | Agmatine |
|  | 0.193 c | 0.099 d | 0.184 c | 0.099 c | 0.15 c | 0.219 c | 0.193 c | 0.191 c | 0.249 c | 0.227 c | 0.314 b | 0.406 a |
| E | Histamine | ẞ -Phenylethylamine | Tyramine | Acetamide | Formamide | Glucuronamide | D,L-Lactamide | D-Glucosamine | D-Galactosamine | D-Mannosamine | N-Acetyl-D-Glucosamine | N-Acetyl-D-Galactosamine |
|  | 0.214 c | 0.189 c | 0.162 c | 0.169 c | 0.251 c | 0.199 c | 0.197 c | 0.275 b | 0.221 c | 0.251 c | 0.265 c | 0.2 c |
| F | N-Acetyl-D-Mannosamine | Adenine | Adenosine | Cytidine | Cytosine | Guanine | Guanosine | Thymine | Thymidine | Uracil | Uridine | Inosine |
|  | 0.207 c | 0.281 b | 0.338 b | 0.284 b | 0.187 c | 0.119 c | 0.381 b | 0.232 c | 0.169 c | 0.193 c | 0.224 c | 0.353 b |
| G | Xanthine | Xanthosine | Uric Acid | Alloxan | Allantoin | Parabanic Acid | D,L- α -Amino-N-Butyric Acid | γ -Amino-N-Butyric Acid | ε -Amino –N-Caproic Acid | D,L- α –Amino Caprylic Acid | δ -Amino –N-Valeric Acid | α -Amino –N-Valeric Acid |
|  | 0.245 c | 0.237 c | 0.284 b | 0.09 d | 0.375 b | 0.16 c | 0.203 c | 0.251 c | 0.164 c | 0.165 c | 0.154 c | 0.15 c |
| H | Ala -Asp | Ala -Gln | Ala -Glu | Ala -Gly | Ala -His | Ala -Leu | Ala -Thr | Gly -Asn | Gly -Gln | Gly -Glu | Gly -Met | Met-Ala |
|  | 0.39 b | 0.281 b | 0.392 b | 0.378 b | 0.341 b | 0.327 b | 0.338 b | 0.326 b | 0.305 b | 0.29 b | 0.229 c | 0.232 c |

Intensity of color change of the plate was monitored at OD_590_ nm

**Table S4:** Number of sequencing read counts of strawberry flower samples

|  | Source | Sample | Total bases | Number of reads |
| --- | --- | --- | --- | --- |
| Strawberry flower in greenhouse | Untreated control | 2-week | 94,763,830 | 314,830 |
|  |  | 4-week | 91,090,426 | 302,626 |
|  |  | 6-week | 83,417,334 | 277,134 |
|  |  | 8-week | 80,470,544 | 267,344 |
|  | L-asparagine | 2-week | 77,825,958 | 258,558 |
|  |  | 4-week | 69,985,510 | 232,510 |
|  |  | 6-week | 73,850,952 | 245,352 |
|  |  | 8-week | 66,117,660 | 219,660 |
|  | L- glutamic acid | 2-week | 81,616,150 | 271,150 |
|  |  | 4-week | 86,002,924 | 285,724 |
|  |  | 6-week | 99,717,086 | 331,286 |
|  |  | 8-week | 90,684,076 | 301,276 |
| Total | | | 995,542,450 | 3,307,450 |

**Table S5:** Number of sequencing read counts of tomato rhizosphere samples

|  | Source | Sample | Total bases | Number of reads |
| --- | --- | --- | --- | --- |
| Tomato | Untreated | 1 week | 69,189,666 | 229,866 |
|  |  | 3 week | 74,860,506 | 248,706 |
|  |  | 7 week | 68,186,455 | 219,856 |
|  |  | 10 week | 67,084,472 | 222,872 |
|  | FOL | 1 week | 56,408,002 | 187,402 |
|  |  | 3 week | 76,346,242 | 253,642 |
|  | L-glutamic acid | 1 week | 75,279,498 | 250,098 |
|  |  | 3 week | 71,562,148 | 237,748 |
|  |  | 7 week | 73,654,157 | 220,458 |
|  |  | 10 week | 63,911,330 | 212,330 |
|  | SP6C4 | 1 week | 72,289,364 | 240,164 |
|  |  | 3 week | 73,535,504 | 244,304 |
|  |  | 7 week | 69,542,532 | 224,651 |
|  |  | 10 week | 67,973,626 | 225,826 |
|  | FOL + L-glutamic acid | 1 week | 69,040,972 | 229,372 |
|  |  | 3 week | 80,931,074 | 268,874 |
|  |  | 7 week | 69,512,565 | 256,354 |
|  |  | 10 week | 65,135,196 | 216,396 |
|  | FOL + SP6C4 | 1 week | 55,116,110 | 183,110 |
|  |  | 3 week | 61,880,784 | 205,584 |
|  |  | 7 week | 63,521,653 | 206,521 |
|  |  | 10 week | 59,352,986 | 197,186 |
|  | FOL + L-glutamic acid + SP6C4 | 1 week | 62,731,410 | 208,410 |
|  |  | 3 week | 68,411,882 | 227,282 |
|  |  | 7 week | 70,542,354 | 213,251 |
|  |  | 10 week | 70,217,882 | 233,282 |
| Total | | | 1,779,218,370 | 5,863,545 |

**Table S6:** GenBank accession numbers for strawberry flower samples determined by Illumina sequencing

| Samples | Untreated | L-asparagine | L-glutamic acid |
| --- | --- | --- | --- |
| SAR | SRR11354704 | SRR11355296 | SRR11355399 |
| BioProject | PRJNA613300 | PRJNA613304 | PRJNA613305 |
| BioSample | SAMN14400492 | SAMN14400493 | SAMN14400506 |

*Replication files were zip at upload format
